# Pediatric Resident Insulin Management Education (PRIME): A Single-Session Workshop Emphasizing Active Learning

**DOI:** 10.15766/mep_2374-8265.11301

**Published:** 2023-02-21

**Authors:** Caroline Schulmeister, Ellen Laves, Jenise Wong, Abby Walch

**Affiliations:** 1 Clinical Fellow, Division of Pediatric Endocrinology, Department of Pediatrics, University of California, San Francisco, School of Medicine; Assistant Professor, Division of Endocrinology, Department of Pediatrics, University of California, Davis, School of Medicine; 2 Associate Professor, Department of Pediatrics, University of California, San Francisco, School of Medicine; 3 Associate Professor, Division of Endocrinology, Department of Pediatrics, University of California, San Francisco, School of Medicine; 4 Clinical Fellow, Division of Pediatric Endocrinology, Department of Pediatrics, University of California, San Francisco, School of Medicine

**Keywords:** Pediatrics, Endocrinology, Pediatric Endocrinology, Insulin, Diabetes, Case-Based Learning

## Abstract

**Introduction:**

Insulin is a high-risk medication, and errors can lead to patient morbidity and mortality. The American Board of Pediatrics recommends that all board-certified pediatricians be able to develop an insulin management plan for patients with diabetes. A needs assessment of pediatric residents revealed low self-efficacy at developing a new subcutaneous insulin plan despite didactic instruction on the topic.

**Methods:**

We created a 90-minute interactive workshop that targeted resident skills in devising subcutaneous insulin plans. Learners engaged in small-group, problem-based learning and peer teaching to promote active learning and participation. We compared self-efficacy and knowledge before and after the intervention using paired *t* tests and evaluated learner satisfaction.

**Results:**

Twenty-eight pediatric interns participated, with 25 completing both the pre- and postworkshop surveys. The primary outcome was self-efficacy (an individual's confidence in the ability to perform a specific task in a given domain). There was a statistically significant improvement in self-efficacy at creating a new subcutaneous insulin plan ( *p* < .001) as well as knowledge ( *p* < .001) after course completion. Learners were highly satisfied with the course, with a mean overall conference quality rating of 4.8 (*SD* = 0.4) based on a 5-point Likert scale (1 = *poor,* 5 = *outstanding*).

**Discussion:**

An interactive workshop employing active learning methods resulted in improved self-efficacy and knowledge in first-year pediatric residents. Future work is needed to determine the impact of this workshop on patient care outcomes.

## Educational Objectives

By the end of this activity, learners will be able to:

1. Differentiate between types of diabetes.
2. Describe the difference between basal and bolus insulin.
3. Recognize patient characteristics that alter insulin need.
4. Create a safe subcutaneous insulin plan.
5. Describe the symptoms of hypoglycemia and how to treat hypoglycemia.

## Introduction

Insulin is a high-risk medication with potential for significant patient morbidity and mortality. This is especially true in children, and insulin ranks as the third most common cause of medication errors in hospitalized children within the United States.^1^ While the American Diabetes Association recommends that children with diabetes receive care from pediatric endocrinologists,^2^ the relative shortage of pediatric endocrinologists^3^ has led to an increasing need for generalists to be prepared to care for children with insulin-dependent diabetes. The American Board of Pediatrics therefore recommends that all board-certified pediatricians be able to develop an insulin management plan for patients with diabetes.^4^ Despite this, resident confidence and perceived competence in insulin prescribing are lacking.^5,6^ A practical and easily integrated insulin curriculum is needed to increase pediatric resident confidence and competence in prescribing this critical medication.

The few existing studies exploring trainee knowledge and comfort with insulin management have demonstrated gaps in trainee insulin management knowledge and perceived competence. A study of more than 2,000 recently graduated medical trainees within the United Kingdom showed that trainees overall lacked confidence in insulin management.^5^ In the United States, a study of internal medicine, family practice, and surgery trainees at one institution showed a significant gap in diabetes knowledge.^6^ Within our own institution, a focused needs assessment performed 4 months prior to implementing this workshop showed that only 32% of learners either somewhat agreed or strongly agreed they would be able to create a new subcutaneous insulin plan. Additionally, only 44% and 16% somewhat agreed or strongly agreed they would be able to calculate an insulin dose and adjust an insulin regimen, respectively.

Although several curricula have been designed to target insulin management in pediatric patients not in diabetic ketoacidosis, most rely on resident self-directed learning to either receive the training or effectively engage during education sessions,^7–10^ are geared towards residents who choose to enroll in an endocrine elective,^11^ or require multiple teaching sessions to administer.^7,11^ It is encouraging that one study showed that pediatric resident insulin teaching can lead to improved inpatient glucose management; however, this success occurred with an 8-week intervention, which may not be practical during residency training.^7^ In addition, reliance on self-directed learning without protected time may be less effective for learners given the demands of residency training. A previous study conducted at the University of California, San Francisco, in 2018 showed improved satisfaction and reported comfort with insulin management in pediatric residents who were given protected time for didactic insulin teaching.^12^ However, our subsequent focused needs assessment demonstrated that further research was needed to identify instructional methods to teach residents insulin management efficiently and effectively.

Pediatric Resident Insulin Management Education (PRIME) aimed to improve insulin-management self-efficacy and knowledge among new pediatric interns during a single-session workshop. The workshop promoted learner engagement by using active learning strategies, including case-based team learning, peer teaching, and peer coaching.^13,14^ We additionally utilized instructor coaching and role-modeling to provide learner scaffolding and encourage learner reflection. To our knowledge, a single-session insulin workshop promoting active learning with insulin management has not been previously published.

## Methods

### Setting and Participants

PRIME targeted University of California, San Francisco, pediatric interns during their first month of training (July/August 2020). The workshop was delivered at an academic half day, during which interns were released from service obligations for focused educational sessions. These half days were developed by the pediatric residency to incorporate core teaching on chosen topics deemed to be important for residents to be exposed to within the first months of residency. We did not ask learners to do prework prior to our session. To prevent service disruptions, PRIME was offered twice, with 50% of the intern class attending each identical session. The University of California, San Francisco, Institutional Review Board deemed the educational session exempt from review.

### Instructional Strategy and Implementation

The workshop promoted learner engagement by utilizing case-based team learning, peer teaching, and peer coaching. Pediatric endocrinology fellows and attendings, as well as the residency's associate program director of curriculum, developed the workshop, which consisted of three main components: (1) a brief overview didactic session; (2) small-group, case-based learning; and (3) peer teaching. Subject matter experts and the associate program director developed relevant learning objectives using the American Board of Pediatrics general content outline^4^ and a targeted needs assessment distributed in April 2020. Due to the COVID-19 pandemic and the need for physical distancing, each 2020 PRIME session was conducted in person in two separate classrooms connected via videoconferencing software. Learners were randomly assigned to one of two classrooms.

Following introductions, the workshop began with a 35-minute presentation delivered to the large group. The presentation briefly reviewed diabetes physiology, types of insulin, and the creation of a subcutaneous insulin plan (Appendix A). During the didactic, the instructor role-modeled insulin calculation by explaining their thought process for a specific case scenario.

Following the didactic session, we randomly assigned learners to groups of three to four individuals. Each group was assigned one of three cases (Appendix B) and instructed to create a safe insulin plan. We developed these cases, which were modeled after patients commonly seen in our hospital. After successful completion of the first case, we provided each group with a more difficult one (the challenge question) that expanded upon topics reviewed during the didactic session. Learners were able to use notes and resources throughout the group activities. One third-year pediatric endocrinology fellow was present in each classroom to coach each group through the case by answering questions, giving feedback, and asking questions to promote reflection. Learners additionally received a calculation handout (Appendix C) to guide their approach to problem-solving.

After all groups had completed both their cases, they rejoined the larger class. At that time, each group presented its patient case and suggested insulin plan. Groups also presented their challenge question and explained their thought process to their peers. During these presentations, the nonpresenting participants were encouraged to comment and ask questions. The facilitators were present throughout the process to help direct the peer teaching and make corrections as needed.

### Facilitators

One third-year pediatric endocrinology fellow with advanced training in medical education delivered the didactic lecture for both PRIME sessions. An additional third-year pediatric endocrinology fellow was present at each of the two 2020 PRIME sessions to assist with the small-group activities and ensure that there was one facilitator in each room. We developed an instructor guide (Appendix D) that detailed the workshop schedule and the small-group cases and explained administration of the group cases. In addition, for each patient case, the instructor guide listed objectives, including those for the challenge question. We distributed the guide to the instructors prior to the session to help them prepare for the workshop. Instructors were not required to attend a training session prior to the workshop.

### Assessment

We developed a survey (Appendix E) to assess the effectiveness of the workshop in achieving the objectives. The survey was administered at the start of the workshop (pretest) and following the conclusion of the final exercise (posttest). The survey included questions, rated by learners on a 5-point Likert scale (1 = *poor,* 5 = *outstanding*), to assess learner ability to perform specific tasks with insulin management. The primary outcome was self-efficacy, defined as an individual's confidence in the ability to perform a specific task in a given domain. The content-based questions were reviewed by faculty and fellows from the Division of Pediatric Endocrinology at the University of California, San Francisco. In addition to this survey, we gave learners the opportunity to evaluate the learning session in a separate evaluation.

### Data Analysis

We used Stata 16.0 (StataCorp) for data analysis and assigned numerical scores to Likert-scale questions. We used tests of proportions—the number of learners who got the question correct out of the total number of responses—to compare the pre- and posttest results for the entire cohort.

## Results

Of the 28 residents who completed PRIME, 25 (89%) finished both the pre- and postworkshop surveys. When measuring self-efficacy, there was an increase in the median score for perceived ability to create a new subcutaneous insulin plan after course completion. Out of 16 knowledge questions, the mean percentage correct increased from 67% preintervention to 91% postintervention. There was a statistically significant increase in the proportion of participants who correctly answered questions assessing knowledge of insulin need while fasting ( *p* = .01) and steroid-induced diabetes physiology ( *p* = .01). In addition, there was a statistically significant increase in the proportion of learners who correctly identified fast-acting insulin ( *p* = .01), long-acting insulin ( *p* = .01), insulin used for infusions ( *p* = .001), and insulin used for insulin pumps ( *p* = .01). Given a clinical case, there was a statistically significant increase in the proportion of learners who correctly calculated the total daily dose of insulin ( *p* = .001), basal insulin ( *p* = .001), insulin sensitivity factor ( *p* = .02), and insulin-to-carbohydrate ratio ( *p* = .006; Table).

**Table.**
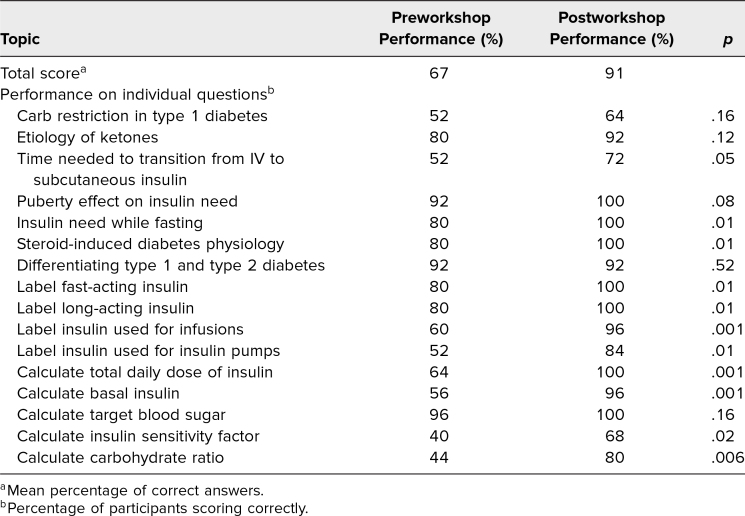
Pre- and Postworkshop Survey Results Among Participants (*n* = 25)

Learners were highly satisfied with the course, with a mean overall conference quality rating of 4.8 (*SD* = 0.4) based on a 5-point Likert scale (1 = *poor,* 5 = *outstanding*).

## Discussion

PRIME utilized active learning strategies such as case-based team learning, peer teaching, and peer coaching to teach insulin management to new pediatric interns. The content was delivered as a discrete session that did not require learner self-directed preparation. The session was well received by residents and resulted in improvement in both self-efficacy and knowledge about insulin management.

Although this workshop was administered to pediatric interns within the first 2 months of starting residency, it could be offered to all years of residency and medical students. The workshop was purposefully designed to be limited to one residency class to encourage team building amongst a new residency class. In addition, we felt that cohorting class years would encourage open participation and limit intimidation that might have occurred if other class levels were present.

Our intervention to deliver insulin education was more forgiving of residents’ schedules compared to those previously described. Despite this, our workshop was shown to have similar improvements in self-efficacy and knowledge as other curricula.^9,11^ As a single 90-minute session, the workshop can be integrated into either an intern orientation or a clinical rotation. It could also be divided into two shorter sessions: a didactic session and then a case-based session. However, further research would be needed to determine the efficacy of such a divided format. In contrast to other described insulin curricula, the workshop did not require any learner prework or self-directed learning, both of which could be challenging during busy clinical rotations.

Medical education has shifted away from the classic pedagogy of an active teacher presentation to passive students. The contemporary student-centered approach prioritizes active learning and improves learner retention.^15^ Unfortunately, learners often rate active learning sessions less positively than passive sessions, which is thought to be due to the increased cognitive effort required during active learning.^16^ In contrast, we successfully utilized active learning techniques in the PRIME sessions and showed improvement in self-efficacy scores while maintaining high learner satisfaction.

We acknowledge potential limitations to this workshop. It was implemented within a single residency program with only 28 participants, which could limit the generalizability of our findings. From an operational standpoint, not all residency programs may have the ability to provide protected time to pediatric residents or the facilitators needed to facilitate small-group work. We developed surveys using content experts but did not perform additional validity or reliability testing. Further work is needed to demonstrate retention of knowledge and feelings of self-efficacy.

It is important to acknowledge that this project has not established causality between our chosen teaching methods and the improvement in self-efficacy and knowledge scores. A randomized control study is needed to compare self-efficacy and knowledge in learners exposed to an active learning session versus a passive learning session. In addition, we ultimately hope to demonstrate that educational interventions employing active learning have positive impacts on patient care as measured by glucose control (i.e., episodes and duration of hyperglycemia and hypoglycemia) in hospitalized patients using insulin and by reduction in medication errors.

In summary, PRIME is a single-session workshop, easily integrated into learners’ schedules, that uses an active learning approach to successfully teach insulin management to pediatric interns. With local adaptation to specific program needs, these techniques can be easily transferred to other settings and curricula.

## Appendices


PRIME Presentation.pptxLearner Cases.docxCalculation Handout.docxInstructor Guide.docxLearner Survey.docx
*All appendices are peer reviewed as integral parts of the Original Publication.*
