# Supplementary material for: Pediatric Resident Insulin Management Education (PRIME): A Single-Session Workshop Emphasizing Active Learning
Source: MedEdPORTAL. 2023 Feb 21;19:11301. doi: 10.15766/mep_2374-8265.11301 (PMC9941370; doi:10.15766/mep_2374-8265.11301)
Supplement: Supplementary file 1 — PRIME Presentation.pptxLearner Cases.docxCalculation Handout.docxInstructor Guide.docxLearner Survey.docx [file mep_2374-8265.11301-s001.zip › B. Learner Cases.docx]

**First Cases:**

Objectives for all cases:

1. Create a safe subcutaneous insulin plan
2. Recognize patient characteristics that alter insulin need

*Group A:* A previously healthy 10kg 18-month-old toddler presents with new onset diabetes, not in DKA. Please propose a reasonable starting subcutaneous insulin plan.

*Group B:* A previously healthy 25kg 8-year-old boy presents in DKA. His gap is closed and he is ready to start subcutaneous insulin. Please propose a reasonable starting subcutaneous insulin plan.

*Group C:* An 80kg 18-year-old female presents with new onset diabetes, not in DKA. She has regular menses and has neck acanthosis on exam. Please propose a reasonable starting subcutaneous insulin plan.

**Second Cases:**

*Group A:*

Objectives:

1. Use an existing subcutaneous insulin regimen to calculate a dose of insulin
2. Incorporate insulin half-life knowledge to avoid “stacking” insulin doses
3. Take into consideration childhood development to determine when an insulin bolus should be given for food

Your patient received his detemir and a dose of aspart for a high BG 2 hours ago. His current blood sugar is 250. He is hungry and would like to eat a pouch. Please calculate how much aspart he should receive, and when it should be given in relationship to when he starts to eat his food?

*Group B:*

Objectives:

1. Describe the differences between a Type 1 diet and a regular diet
2. Diagnose DKA
3. Develop a hypoglycemia treatment plan

Part 1: Your patient is in the pediatric ward and you place him on a “Type 1 diet.” How does a “Type 1 diet” differ from a typical pediatric diet?

Part 2: He is successfully discharged home and then returns to the ED the following month with emesis and diarrhea. In the ED he has a blood glucose of 69 with a pH of 7.4 and small ketones. Is he in DKA? How should you manage his hypoglycemia?

*Group C:*

Objectives:

1. Describe clinical features that can help differentiate between different types of diabetes
2. Describe lab tests that can be used to differentiate between type 1 and type 2 diabetes.
3. Determine how insulin should be altered during times of fasting

Part 1: You suspect that your patient has type 2 diabetes. What clinical features could help you differentiate between new onset type 1 and type 2 diabetes? What lab tests can you get to help differentiate between type 1 and type 2 diabetes?

Part 2: Your patient will be NPO (nothing by mouth) overnight. How would you adjust this patient’s insulin regimen overnight?
