# Supplementary material for: Pediatric Resident Insulin Management Education (PRIME): A Single-Session Workshop Emphasizing Active Learning
Source: MedEdPORTAL. 2023 Feb 21;19:11301. doi: 10.15766/mep_2374-8265.11301 (PMC9941370; doi:10.15766/mep_2374-8265.11301)
Supplement: Supplementary file 1 — PRIME Presentation.pptxLearner Cases.docxCalculation Handout.docxInstructor Guide.docxLearner Survey.docx [file mep_2374-8265.11301-s001.zip › C. Calculation Handout.docx]

Calculating Insulin: Cheat Sheet

Step 1: Calculate Total Daily Dose (TDD):

Pre Pubertal Post Pubertal

0.5 units/kg/day 0.7 units/kg/day

Step 2: Calculate Basal:

50% of TDD

Step 3: Calculate Bolus:

Carbohydrate Factor: 500/TDD

High BG Factor: 1800/TDD

Step 4: Establish a Target BG:

Safe Target =120
