# Supplementary material for: Pediatric Resident Insulin Management Education (PRIME): A Single-Session Workshop Emphasizing Active Learning
Source: MedEdPORTAL. 2023 Feb 21;19:11301. doi: 10.15766/mep_2374-8265.11301 (PMC9941370; doi:10.15766/mep_2374-8265.11301)
Supplement: Supplementary file 1 — PRIME Presentation.pptxLearner Cases.docxCalculation Handout.docxInstructor Guide.docxLearner Survey.docx [file mep_2374-8265.11301-s001.zip › D. Instructor Guide.docx]

**PRIME Instruction Guide**

**Brief Introduction**

The Pediatric Resident Insulin Management Education (PRIME) curriculum is aimed to improve insulin-management self-efficacy and knowledge among new pediatric interns during a single-session workshop. This workshop promotes learner engagement by using active learning strategies including case-based team learning, peer teaching, and peer coaching. Instructor coaching and role-modeling should be used to provide learner scaffolding and to encourage learner reflection.

**Pre-curriculum Facilitatory Instruction:**

Familiarized yourself with the materials provided. You will need a yogurt pouch (or substitute carbohydrate containing food) for one of the cases. It may be useful to print out the learner cases and place onto notecards to handout to learners during the session.

**Learning Objectives:**

At the end of the curriculum learners should be able to:

- Differentiate between different types of diabetes mellitus (dm)
- Describe the difference between basal and bolus insulin
- Recognize patient characteristics that alter insulin need
- Create your own safe subcutaneous insulin plan
- Describe the symptoms of hypoglycemia and how to treat hypoglycemia

**Curriculum Schedule (90 min total):**

-35 min: Didactic Lecture (Appendix A)

-40 min: Interactive Cases

-5min: 1^st^ case

-10min: 2^nd^ case

-24min: peer teaching (8min per team)

-15 min: Post-Workshop Survey, Questions/Close

**Implementation Recommendations**

The curriculum was initially intended to be used for first year pediatric residents, however, it could be used for any year of training. The curriculum consists of 3 main components: 1) a brief overview didactic session, 2) small group case-based learning, and 3) peer teaching.

Following introductions, the workshop begins with a 35-minute presentation delivered to the large group (Appendix A). During the didactic session, the instructor should model expert cognitive thought process by doing a verbal think aloud while solving a commonly encountered problem in diabetes management. Following the didactic session, learners should be divided into three groups. Each group should be assigned to one of three cases (Appendix B). For the first case, each group will be given a patient description and will be asked to develop an appropriate subcutaneous insulin plan for that patient. A calculation handout (Appendix C) can be handed out to learners to help them go through the process of creating a new insulin plan. Each group should review their answers with one of the facilitators to confirm dosing prior to moving on to the next case. Instructors should move around the classroom and assist groups with questions as needed.

After successfully creating an insulin plan, each group will be given the second case (Appendix B) which will vary in the clinical question being explored. Each group will keep the patient they were given for the first case and apply their second case with this initial patient in mind. Groups will need to successfully complete the second case and review their answer with an instructor. When answering the “challenge case” the learners should be encouraged to share their thought process in detail for further review, discussion, and analysis. Instructors should provide scaffolding by verbalizing their thought processes when approaching each problem with a focus on comparing the “challenge case” to the less complex question, and describing how they consider the interactive elements. It is important for instructors to ensure that learners understand the “challenge question” to their case prior to peer teaching.

Once all of the groups have successfully completed their second case, each group will have 8 min to teach their peers about the lessons they learned from the case. During the peer teaching, each group should first read out loud their patient blurb from the first case, and then read out loud the scenario and question from the second case. Learners should present their answer to the question from the second case with an emphasis on patient factors that guided their decisions. You should welcome learners to use metacognition skills during their presentation by discussing mistakes they may have made when thinking about each scenario and how they were able to come to the correct answer.

After completion of the curriculum, a Learner Survey (Appendix E) can be distributed to learners to assess curriculum effectiveness.
