# Supplementary material for: Pediatric Resident Insulin Management Education (PRIME): A Single-Session Workshop Emphasizing Active Learning
Source: MedEdPORTAL. 2023 Feb 21;19:11301. doi: 10.15766/mep_2374-8265.11301 (PMC9941370; doi:10.15766/mep_2374-8265.11301)
Supplement: Supplementary file 1 — PRIME Presentation.pptxLearner Cases.docxCalculation Handout.docxInstructor Guide.docxLearner Survey.docx [file mep_2374-8265.11301-s001.zip › E. Learner Survey.docx]

**Appendix E. Learner Survey**

1. If confronted with a child with suspected diabetes, I know how to confirm a diagnosis of diabetes:
   1. Strongly agree
   2. Somewhat agree
   3. Neutral
   4. Somewhat disagree
   5. Strongly disagree
2. If confronted with a child with a new diagnosis of diabetes, I know how to create a new subcutaneous insulin plan:
   1. Strongly agree
   2. Somewhat agree
   3. Neutral
   4. Somewhat disagree
   5. Strongly disagree
3. If confronted with a child with known diabetes, I know how to use an existing subcutaneous insulin plan to calculate an insulin dose:
   1. Strongly agree
   2. Somewhat agree
   3. Neutral
   4. Somewhat disagree
   5. Strongly disagree

**Please answer the following questions to the best of your ability:**

1. A Type 1 Diabetes Diet restricts the amount of carbohydrates allowed.
   1. True
   2. False
2. You are admitting a patient with DKA. The etiology of their ketones is:
   1. Prolonged elevated glucose levels
   2. Insufficient insulin
3. How would you transition a patient from insulin infusion to subcutaneous insulin?
   1. Stop the insulin infusion once the anion gap is closed, then give subcutaneous insulin
   2. Stop the insulin infusion at the same time you give subcutaneous insulin
   3. Stop the insulin infusion after overlapping with subcutaneous insulin for at least 1 hour
4. Insulin units per kilogram requirements are different in pre-pubertal versus pubertal children.
   1. True
   2. False
5. A patient with Type 1 diabetes is NPO at midnight for a surgery. Her long acting insulin should be discontinued overnight.
   1. True
   2. False
6. A patient has steroid induced diabetes. From a pathophysiology standpoint, their diabetes most closely resembles:
   1. Type 1 DM
   2. Type 2 DM
7. An obese patient with acanthosis and a strong family history of type 2 diabetes was recently diagnosed with diabetes. No further work up is needed to differentiate between type 1 and type 2 diabetes in this patient.
   1. True
   2. False
8. Detemir is which type of insulin:
   1. Long acting
   2. Short acting
9. Aspart is which type of insulin:
   1. Long Acting
   2. Short Acting
10. What insulin do we typically use for intravenous infusion?
    1. lispro (Humalog)
    2. Regular
    3. glargine (Lantus)
11. What type of insulin do we typically use in insulin pumps?
    1. Short Acting
    2. Intermediate Acting
    3. Long Acting

**Practice Case:**

A 40kg previously healthy 11 yr old girl with breast budding presented in DKA and new-onset diabetes mellitus. Her anion gap has closed on the insulin infusion, and she is ready to be transitioned to a subcutaneous insulin regimen. She is hungry and wants to eat breakfast. Please select a reasonable starting regimen in the 5 questions below:

1. Total Daily Dose of Insulin
   1. 12
   2. 25
   3. 100
2. Basal Insulin (assume given once daily)
   1. 12
   2. 25
   3. 100
3. Blood Glucose Target
   1. 12
   2. 25
   3. 100
4. High Blood Glucose Factor
   1. 12
   2. 25
   3. 100
5. Carbohydrate Factor
   1. 12
   2. 25
   3. 100
