# Supplementary material for: Pediatric Resident Insulin Management Education (PRIME): A Single-Session Workshop Emphasizing Active Learning
Source: MedEdPORTAL. 2023 Feb 21;19:11301. doi: 10.15766/mep_2374-8265.11301 (PMC9941370; doi:10.15766/mep_2374-8265.11301)
Supplement: Supplementary file 1 — PRIME Presentation.pptxLearner Cases.docxCalculation Handout.docxInstructor Guide.docxLearner Survey.docx [file mep_2374-8265.11301-s001.zip › A. PRIME Presentation.pptx]

## Slide 1
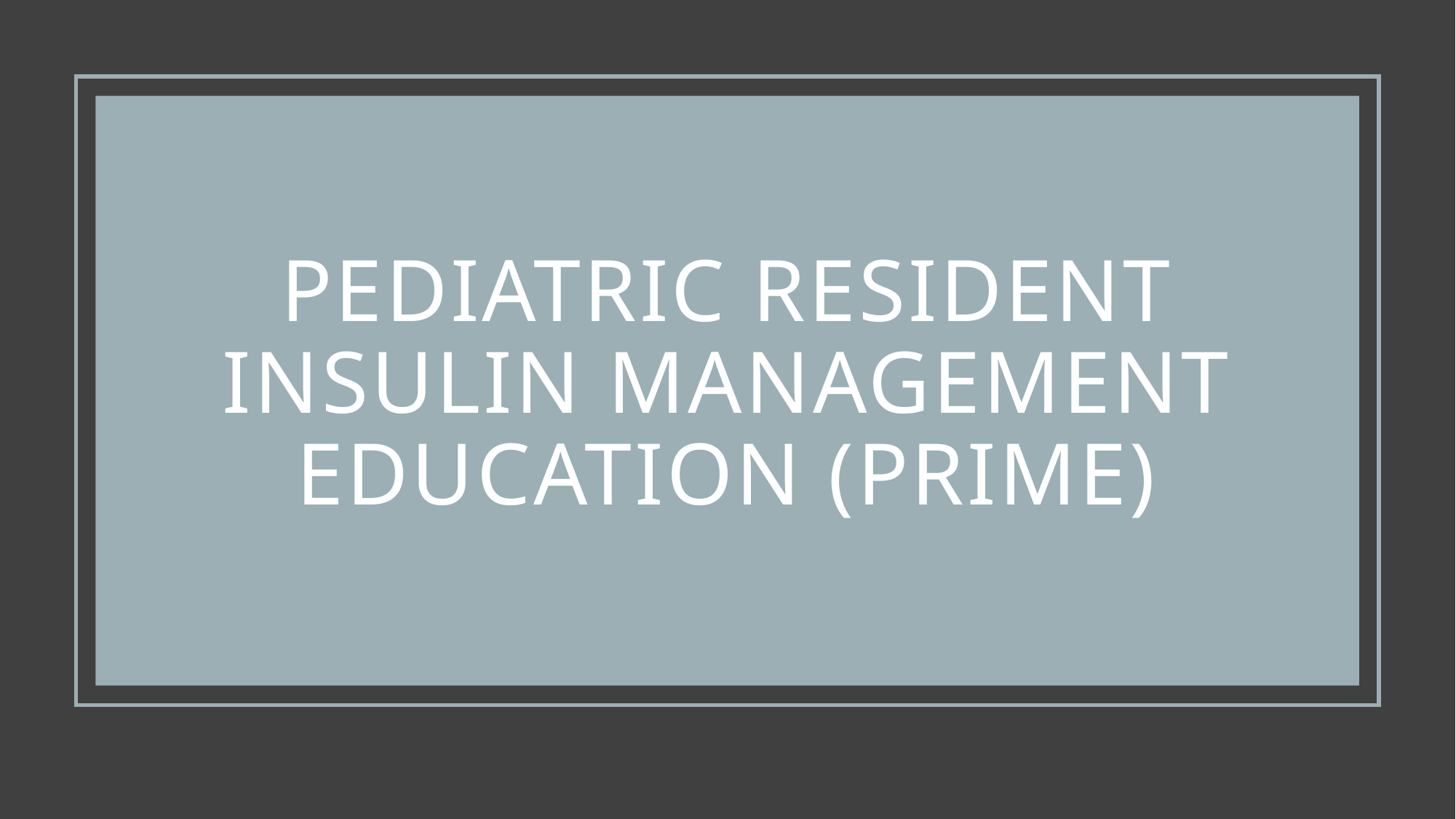

# Pediatric Resident Insulin Management education (PRIME)

## Slide 2
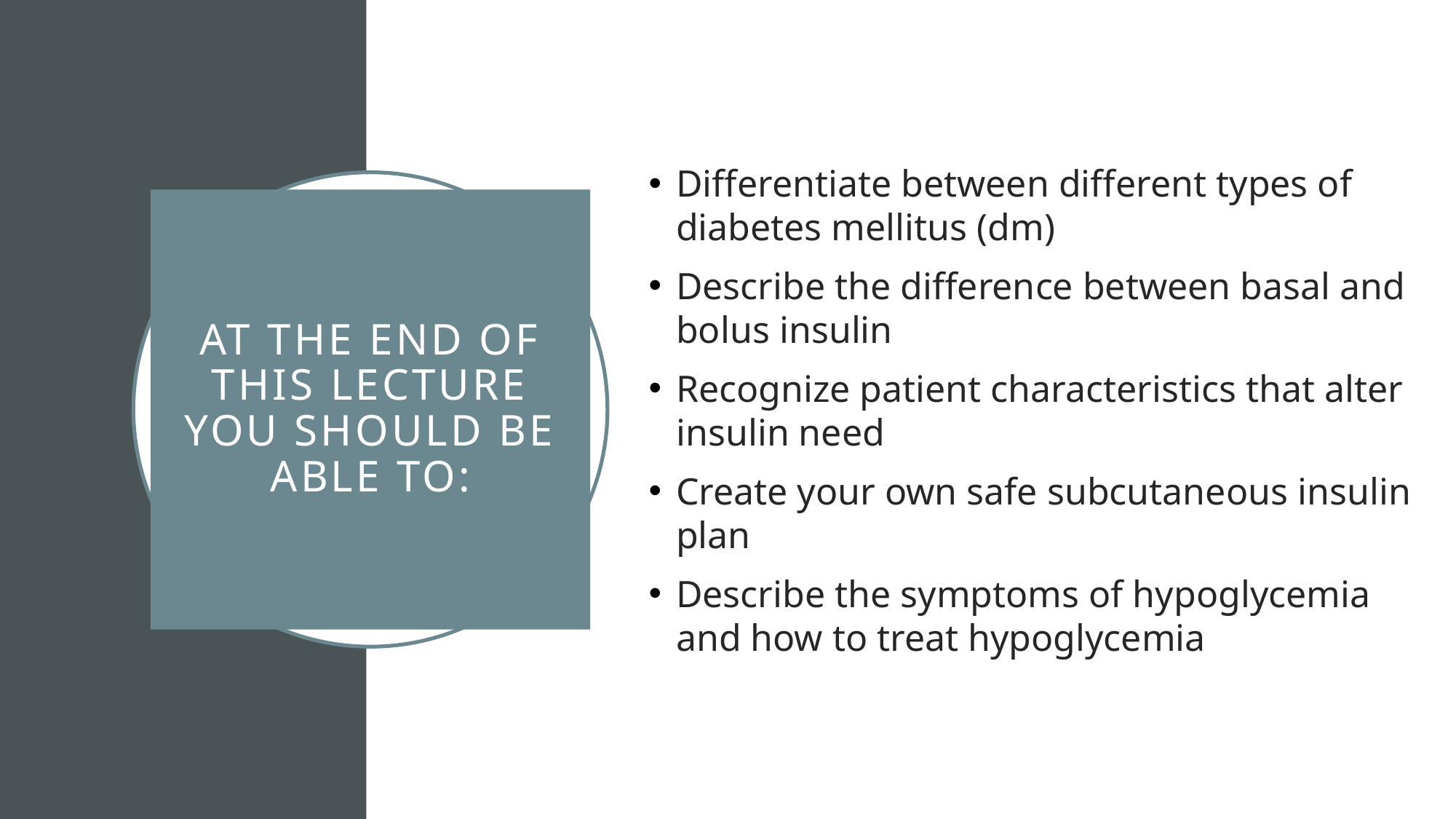

Differentiate between different types of diabetes mellitus (dm)
Describe the difference between basal and bolus insulin
Recognize patient characteristics that alter insulin need
Create your own safe subcutaneous insulin plan
Describe the symptoms of hypoglycemia and how to treat hypoglycemia
# At the end of this lecture you should be able to:

## Slide 3
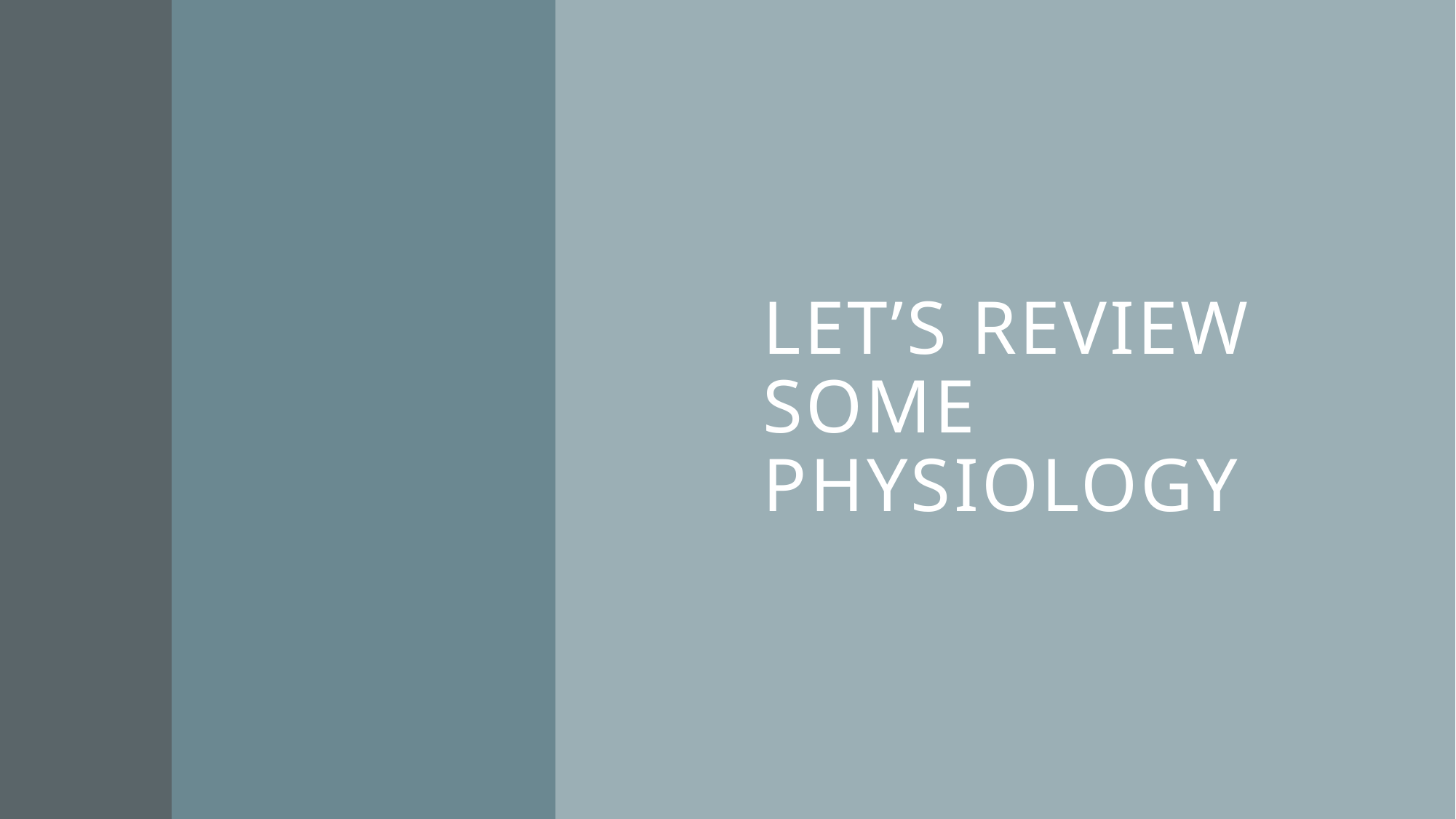

# Let’s review some physiology

## Slide 4
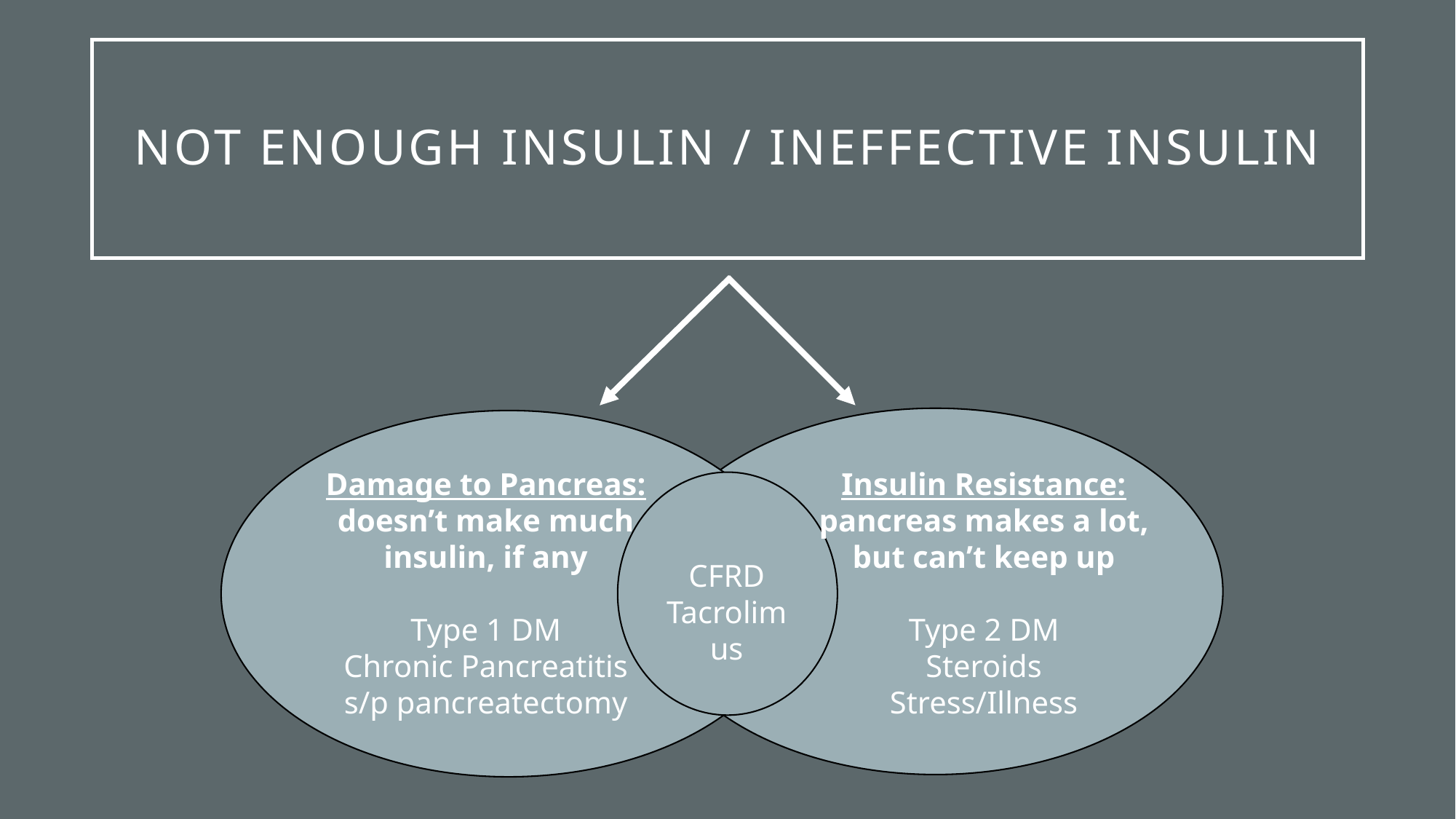

# Not enough insulin / ineffective insulin
Damage to Pancreas: doesn’t make much insulin, if any
Type 1 DM
Chronic Pancreatitis
s/p pancreatectomy
Insulin Resistance: pancreas makes a lot, but can’t keep up
Type 2 DM
Steroids
Stress/Illness
CFRD
Tacrolimus

## Slide 5
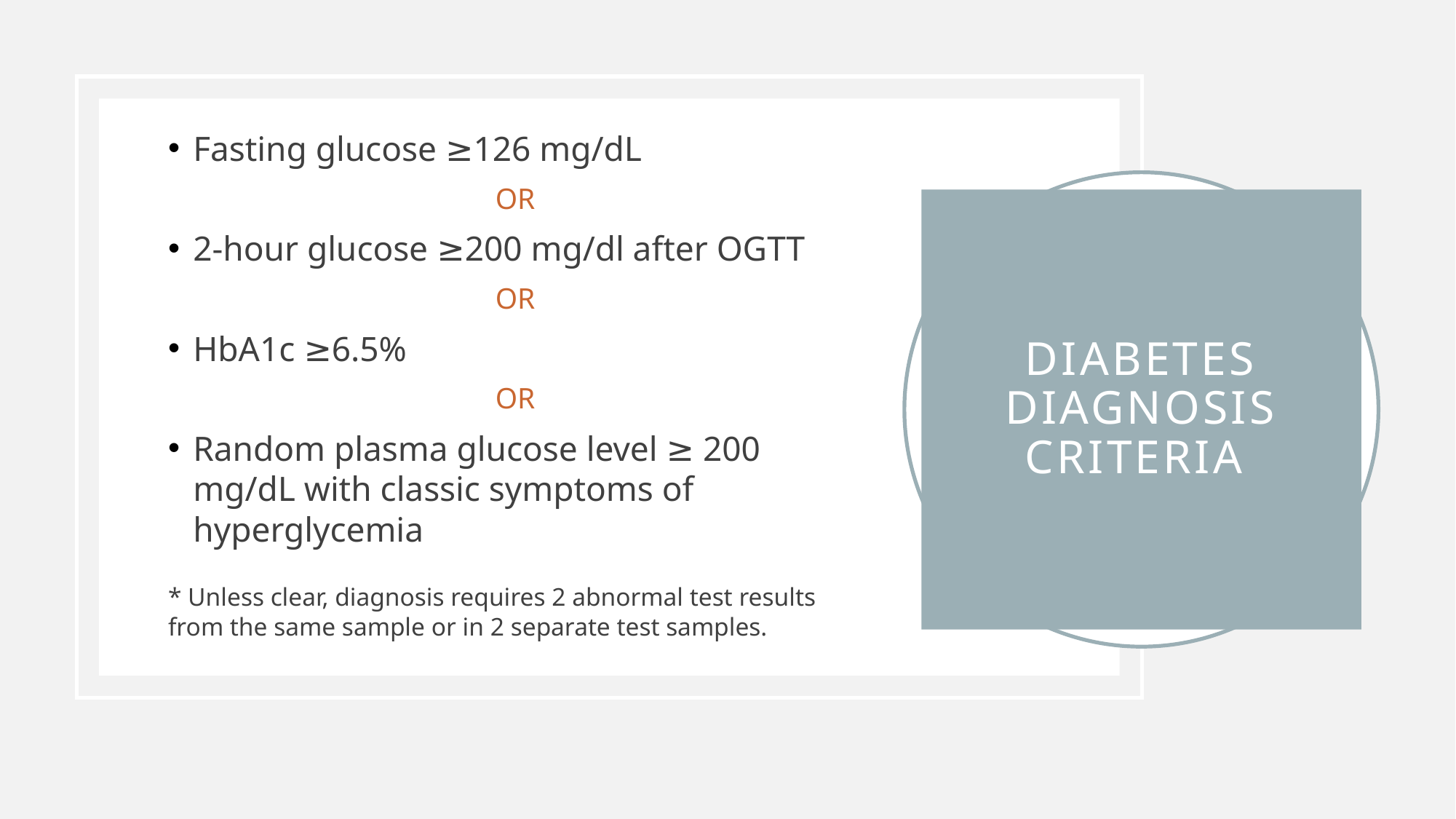

Fasting glucose ≥126 mg/dL
OR
2-hour glucose ≥200 mg/dl after OGTT
OR
HbA1c ≥6.5%
OR
Random plasma glucose level ≥ 200 mg/dL with classic symptoms of hyperglycemia
* Unless clear, diagnosis requires 2 abnormal test results from the same sample or in 2 separate test samples.
# Diabetes Diagnosis Criteria

## Slide 6
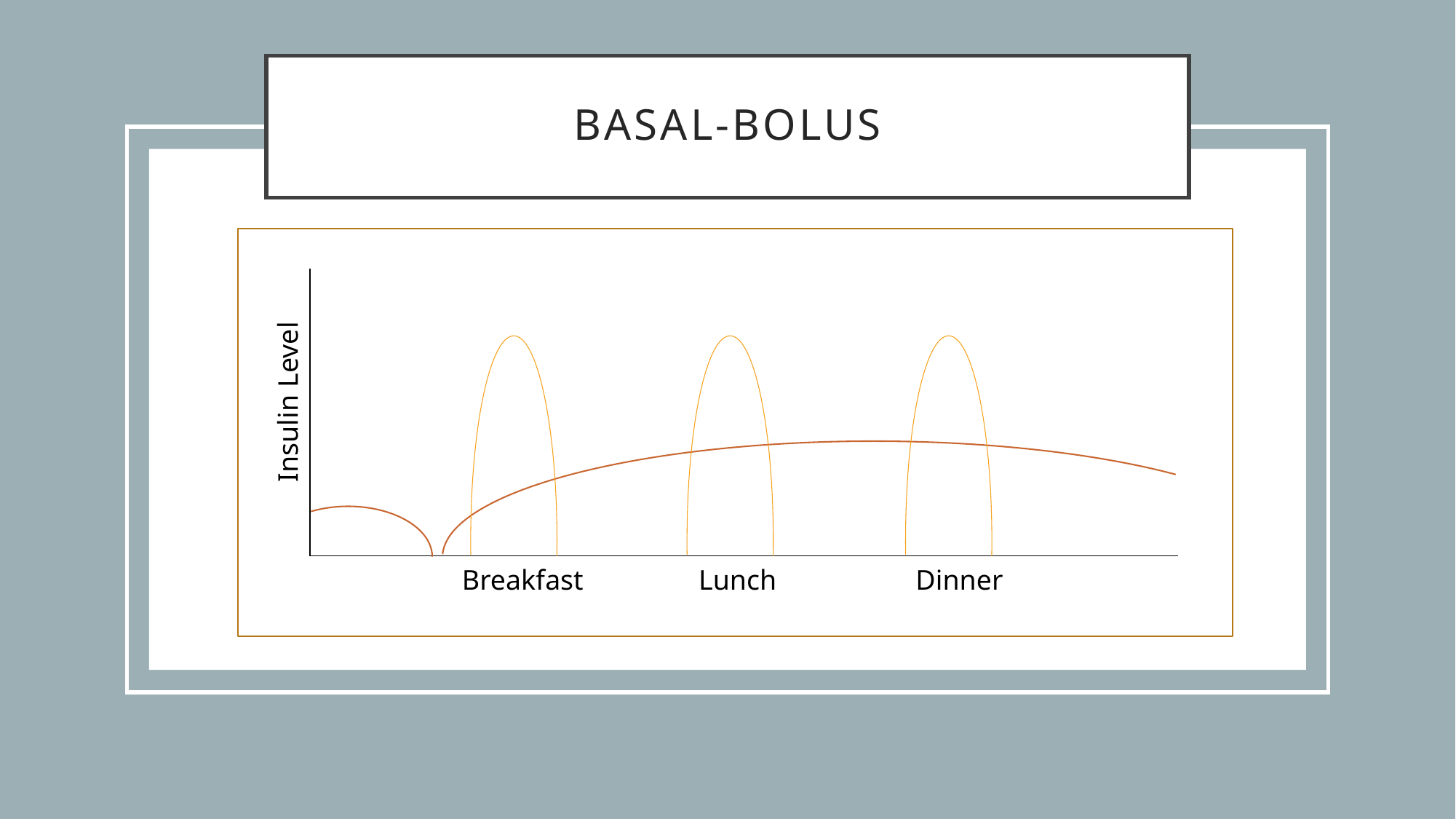

# Basal-bolus
Insulin Level
Lunch
Dinner
Breakfast

## Slide 7
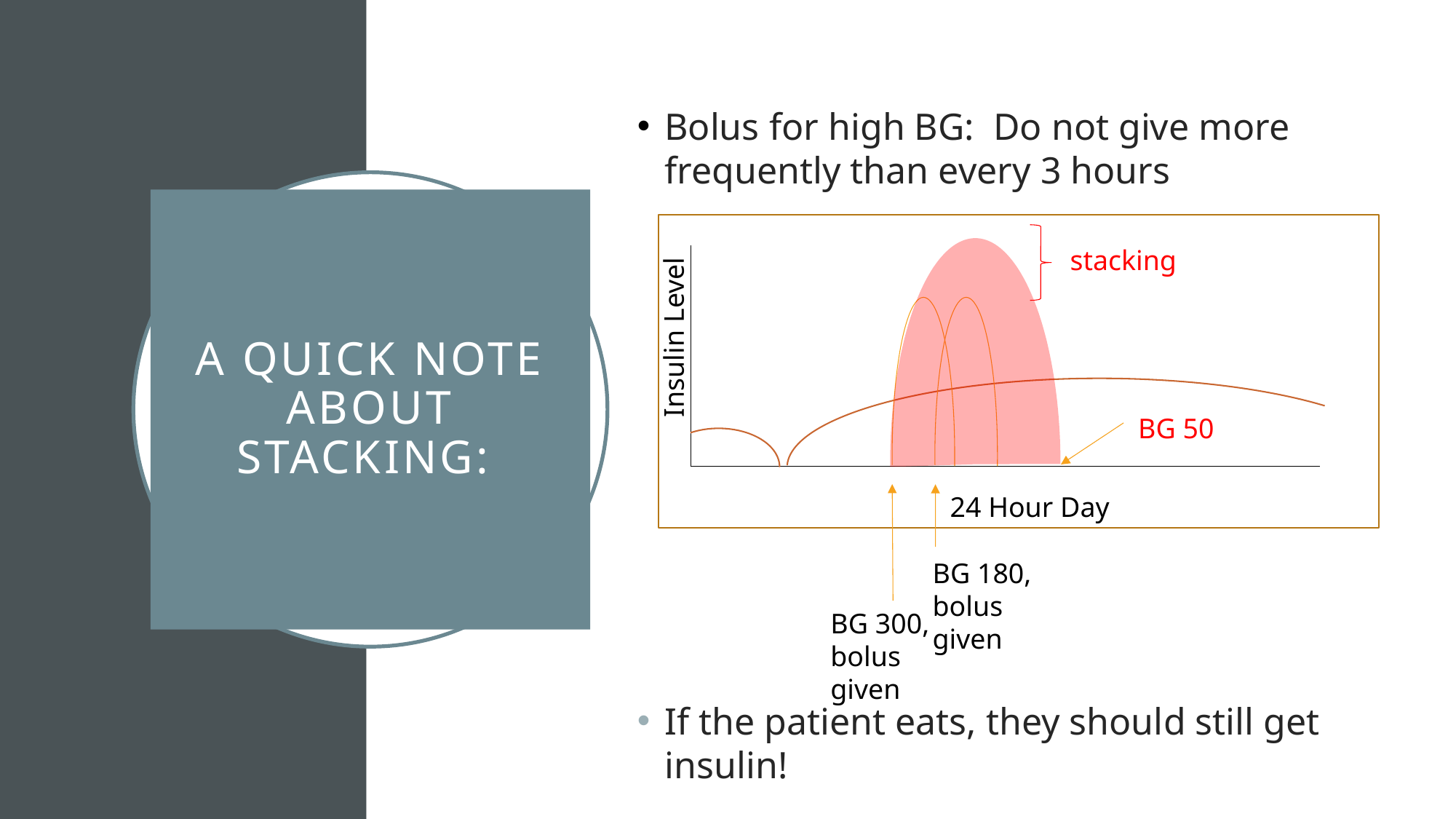

Bolus for high BG: Do not give more frequently than every 3 hours
# A quick note about stacking:
Insulin Level
24 Hour Day
stacking
BG 50
BG 180, bolus given
BG 300, bolus given
If the patient eats, they should still get insulin!

## Slide 8
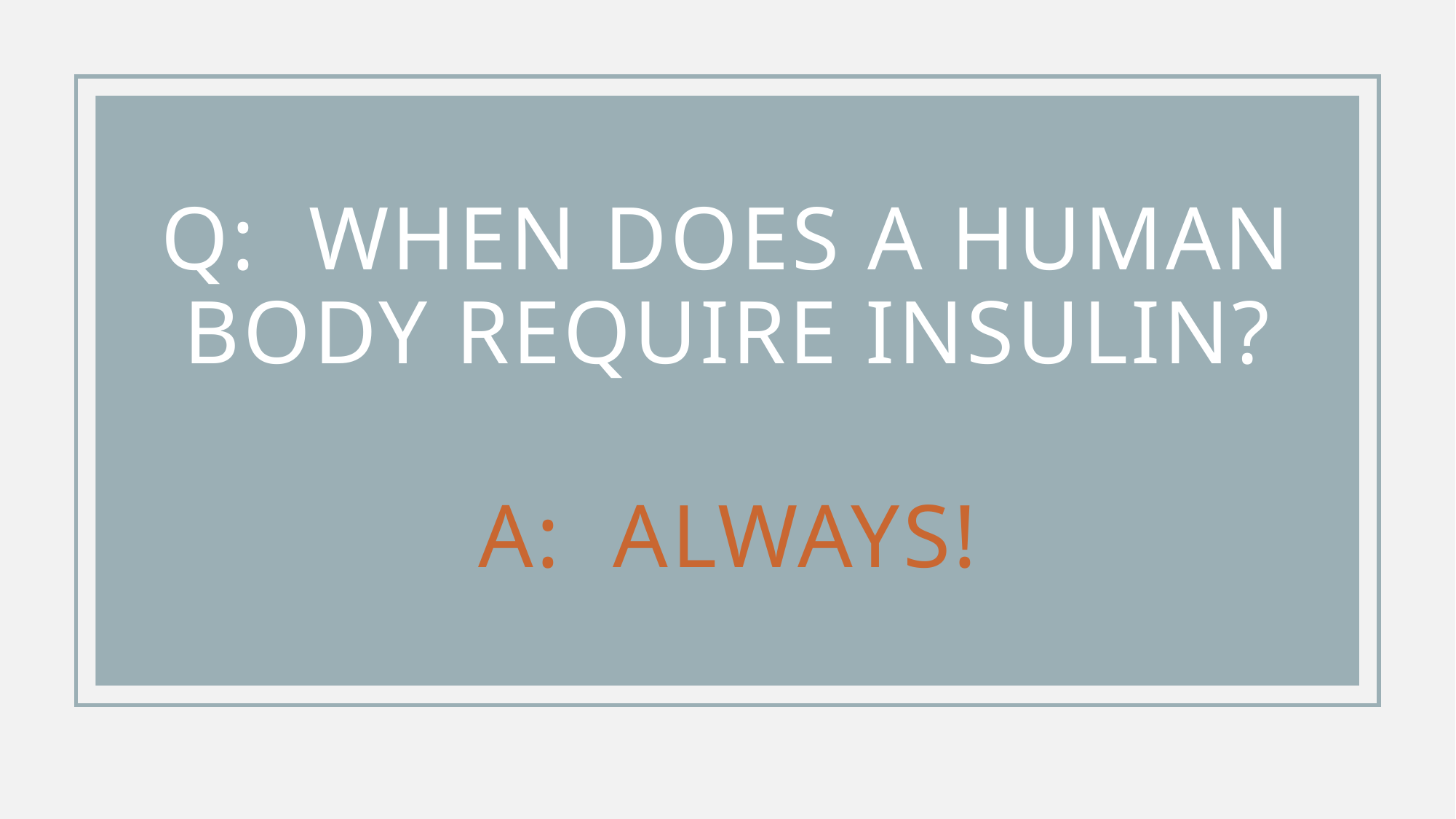

Q: When does a human body require insulin?
A: ALWAYS!

## Slide 9
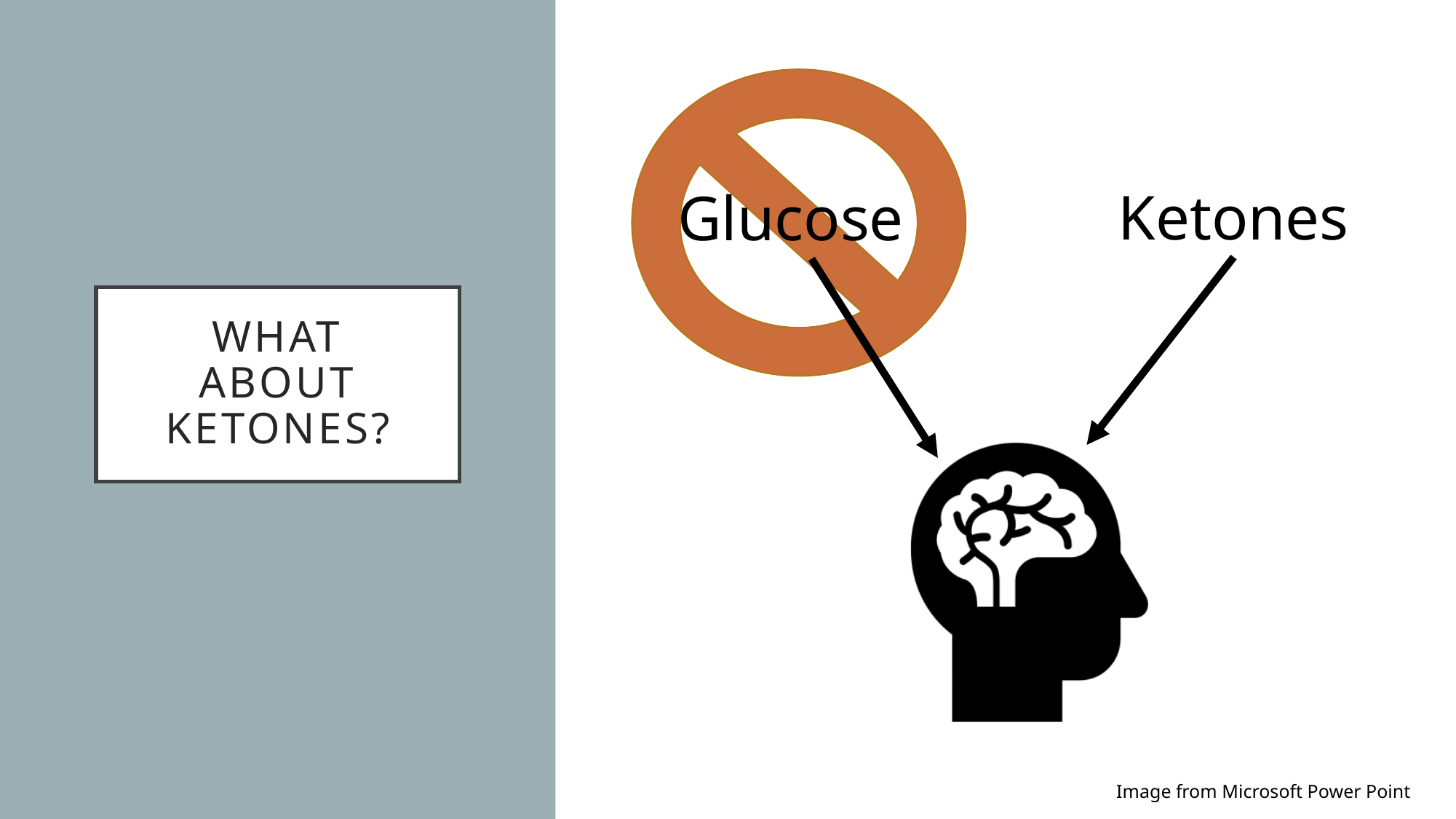

Ketones
Glucose
# What About Ketones?
Image from Microsoft Power Point

## Slide 10
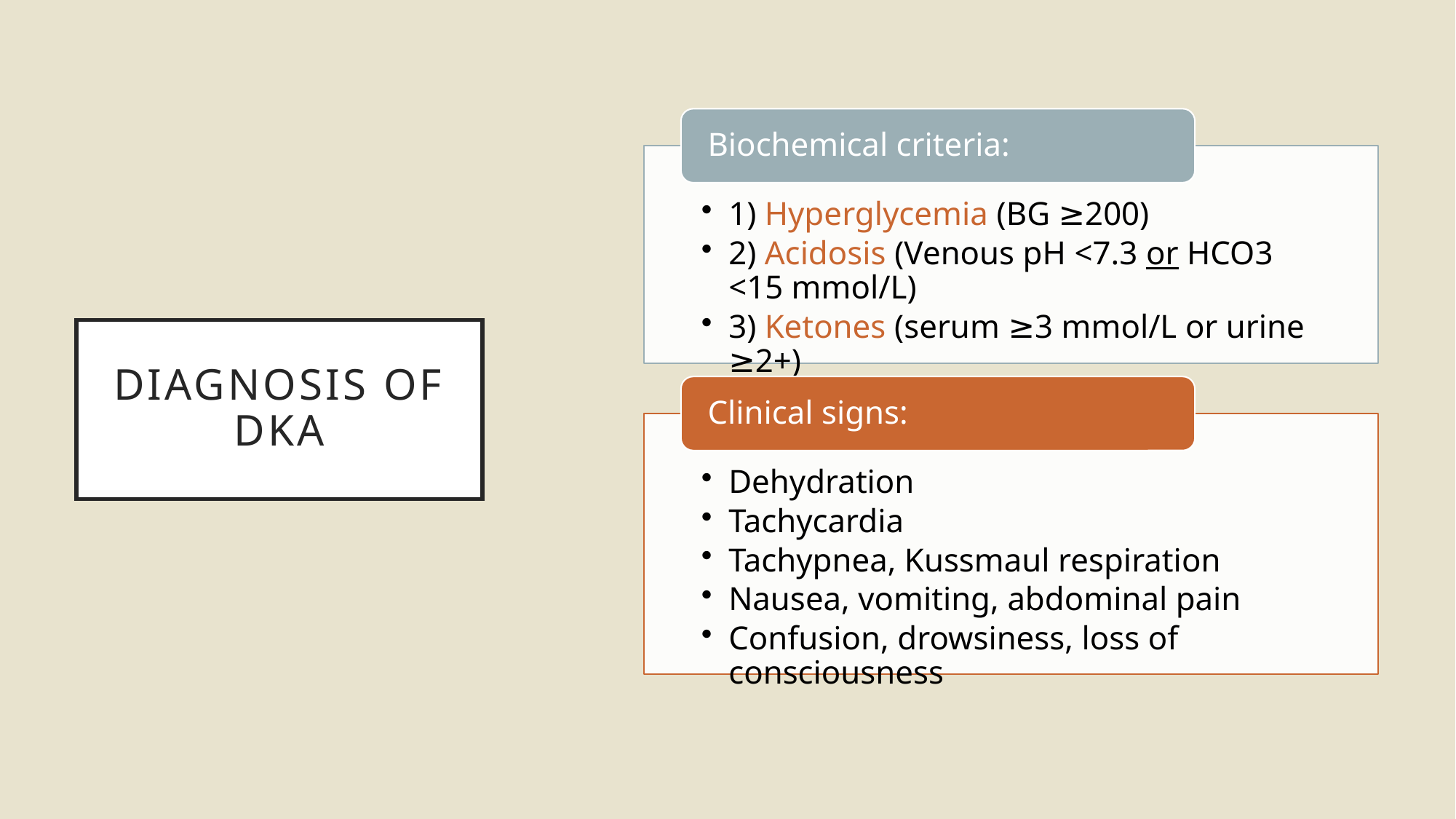

# Diagnosis of DKA

## Slide 11
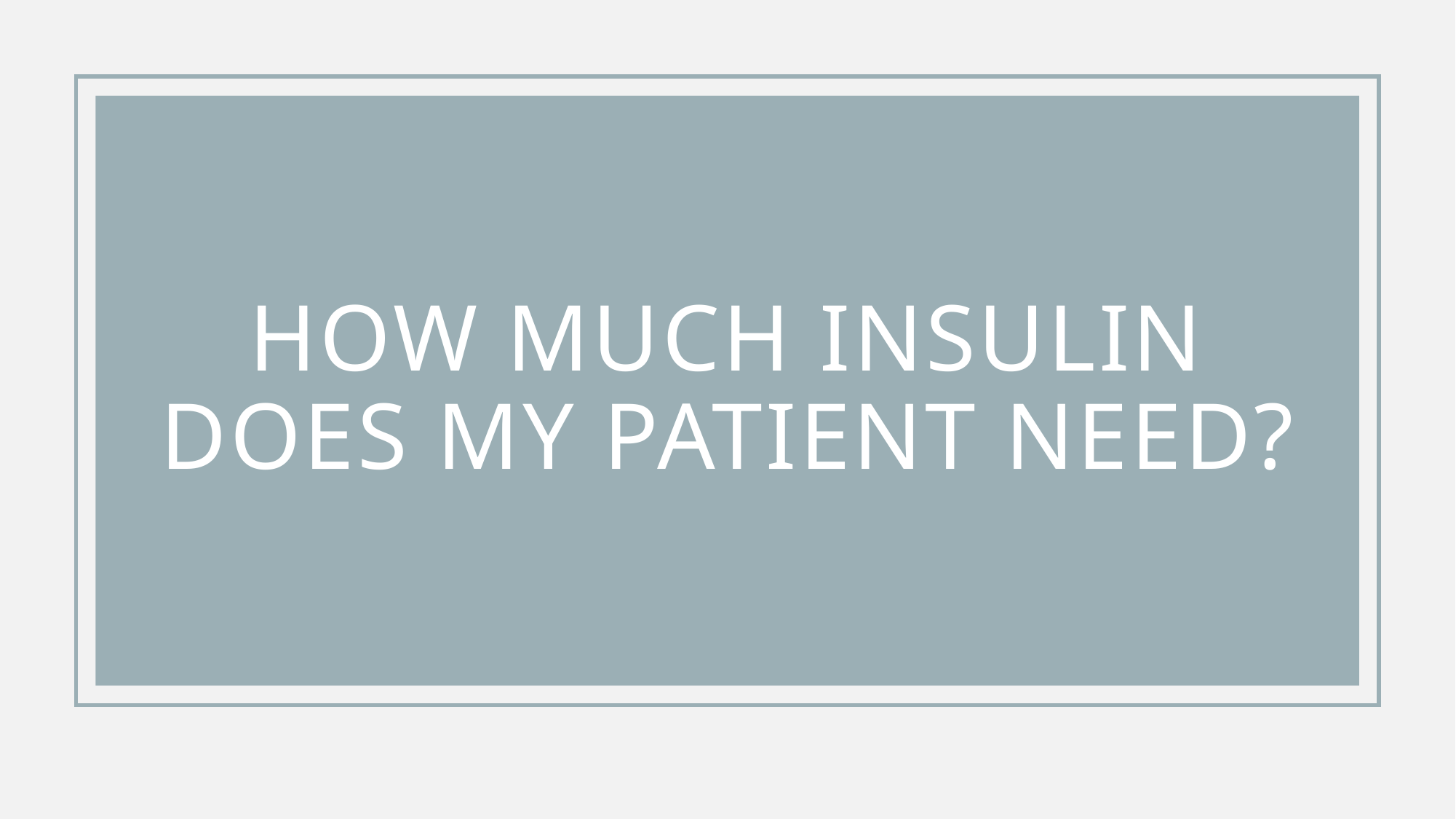

# How Much Insulin Does My Patient Need?

## Slide 12
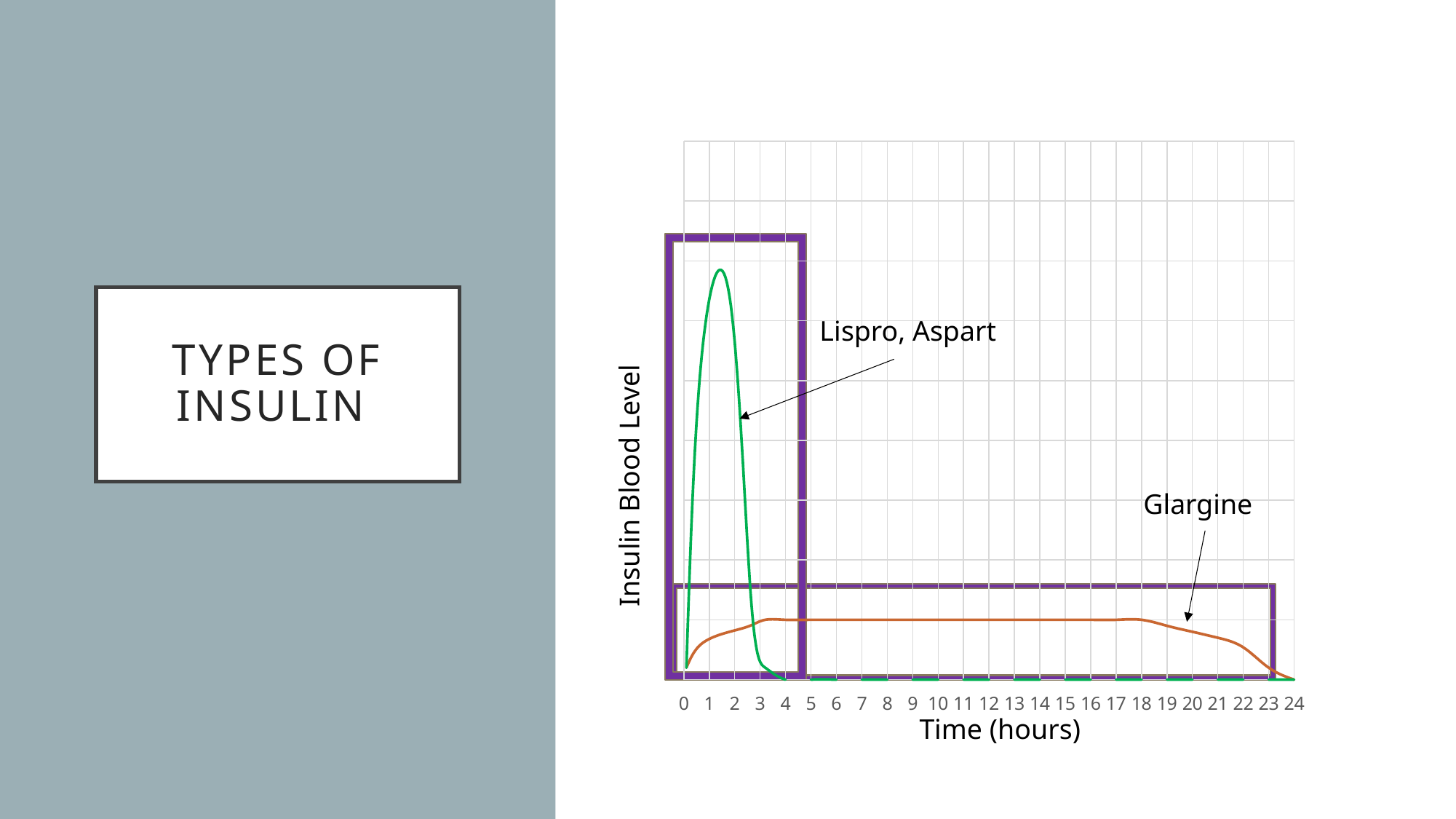

### Chart
| Category | Y-Values | Y-Values2 |
|---|---|---|
# Types of Insulin
Lispro, Aspart
Insulin Blood Level
Glargine
Time (hours)

## Slide 13
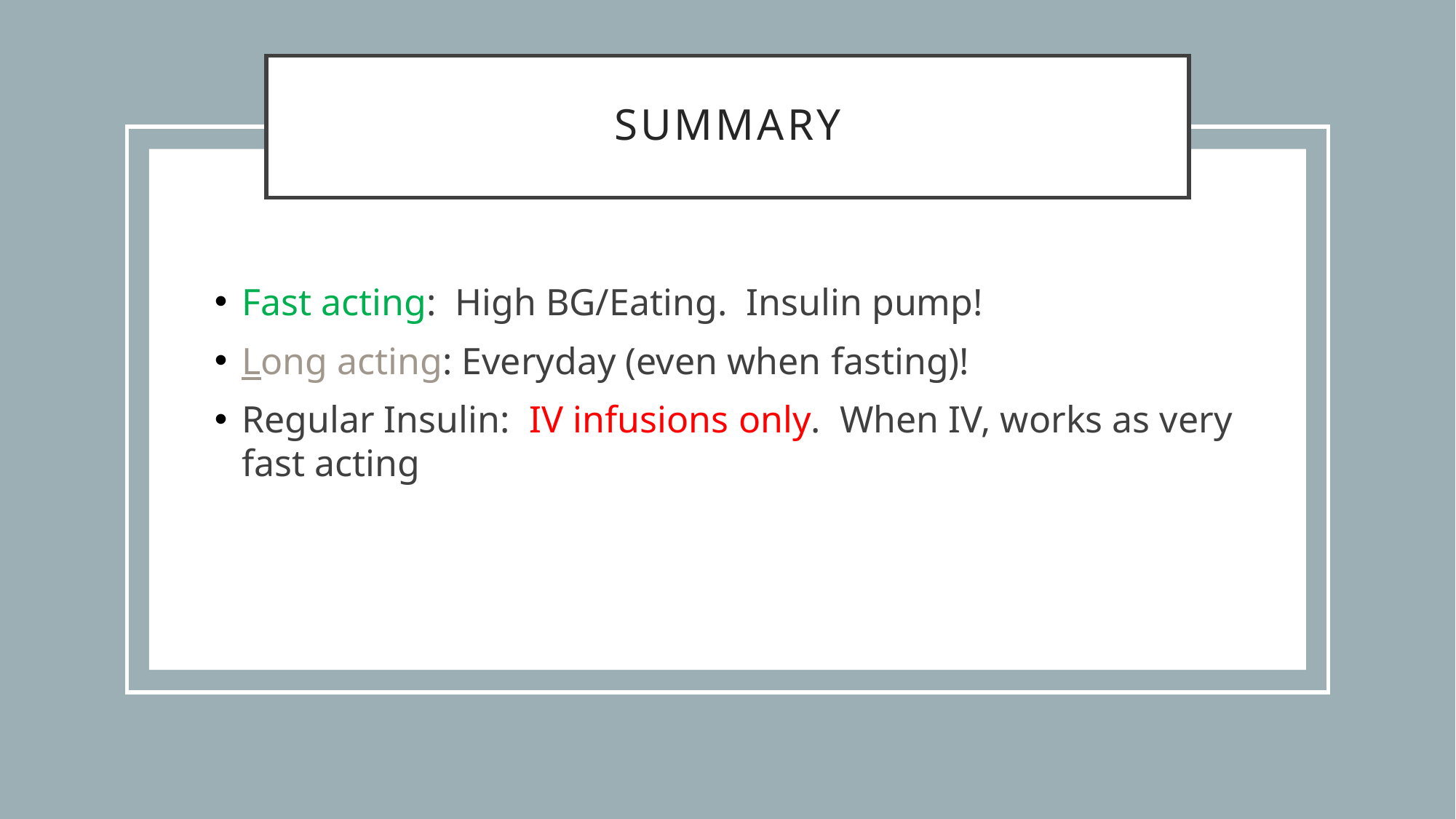

# Summary
Fast acting: High BG/Eating. Insulin pump!
Long acting: Everyday (even when fasting)!
Regular Insulin: IV infusions only. When IV, works as very fast acting

## Slide 14
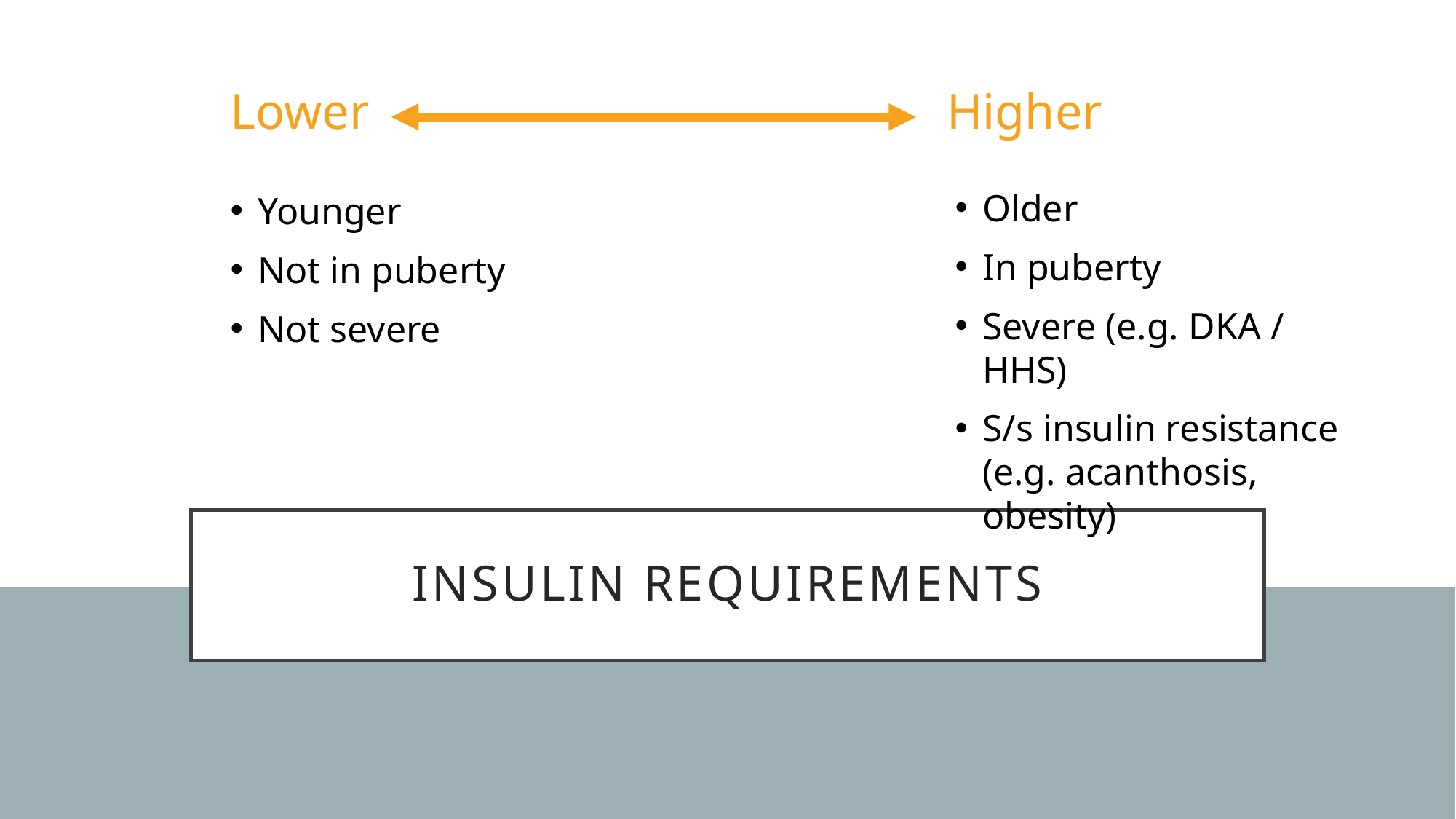

Lower
Higher
Older
In puberty
Severe (e.g. DKA / HHS)
S/s insulin resistance (e.g. acanthosis, obesity)
Younger
Not in puberty
Not severe
# insulin requirements

## Slide 15
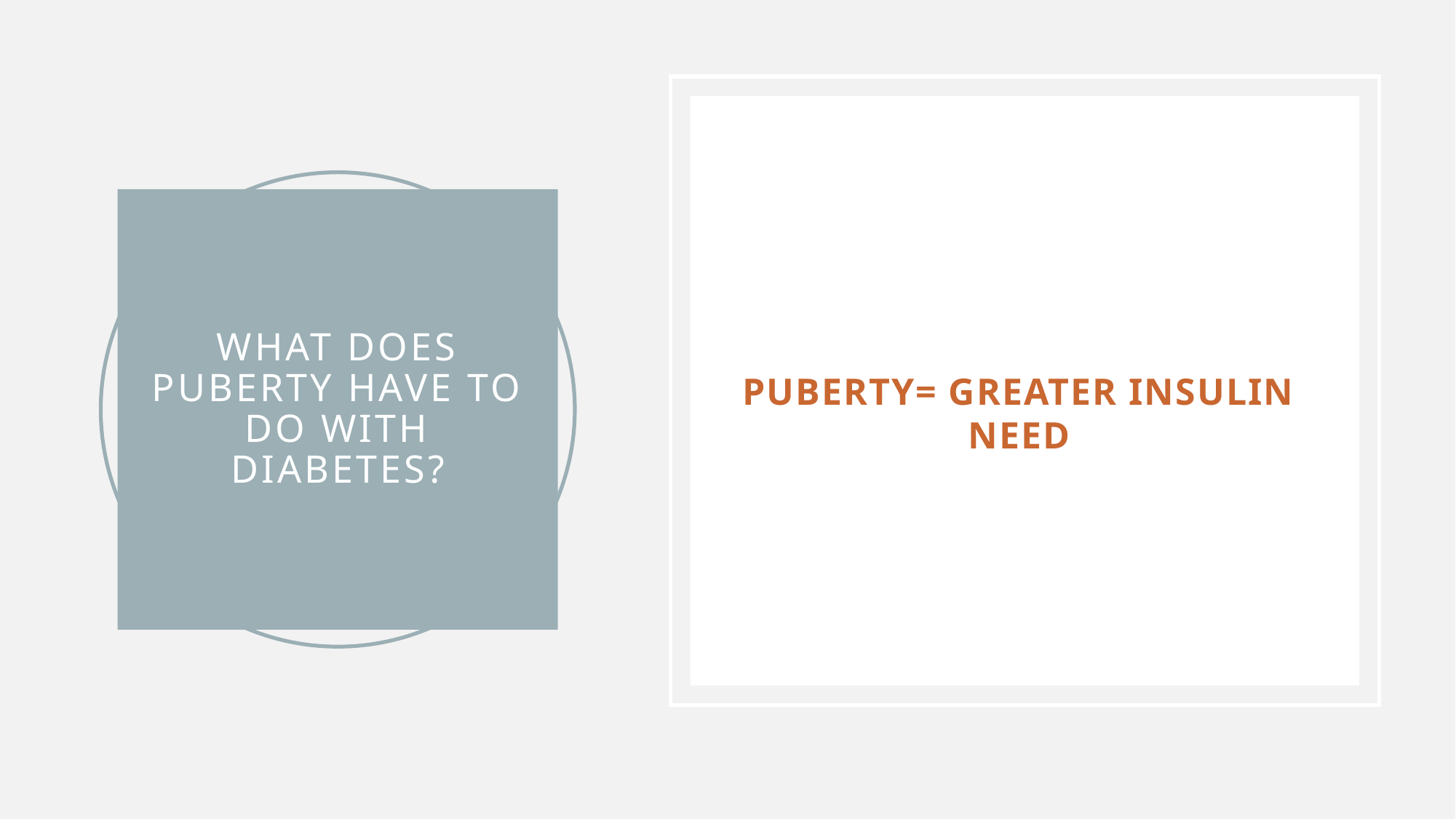

# What does puberty have to do with Diabetes?
PUBERTY= GREATER INSULIN NEED

## Slide 16
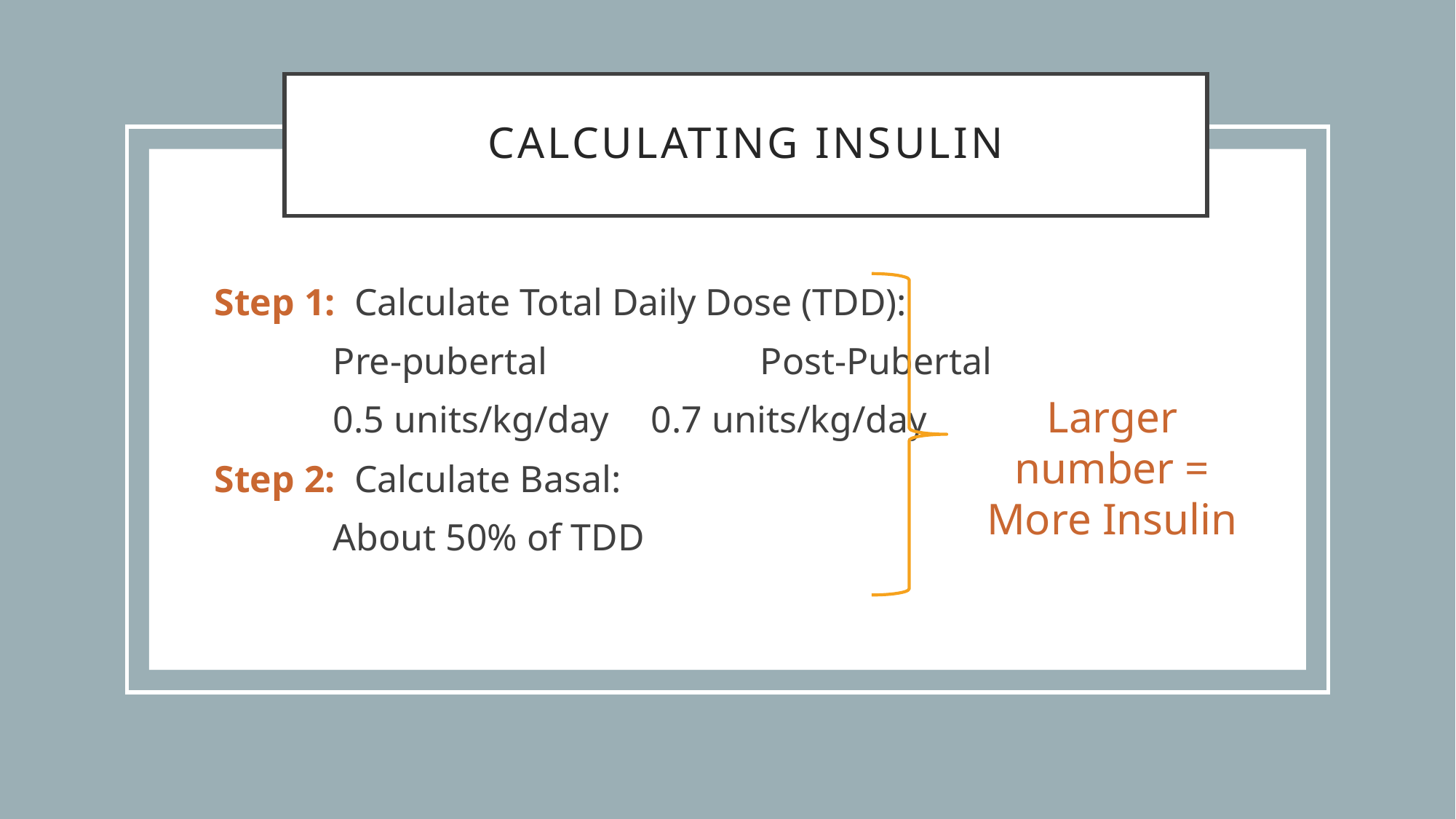

Calculating insulin
Step 1: Calculate Total Daily Dose (TDD):
	 Pre-pubertal		Post-Pubertal
	 0.5 units/kg/day	0.7 units/kg/day
Step 2: Calculate Basal:
	 About 50% of TDD
Larger number = More Insulin

## Slide 17
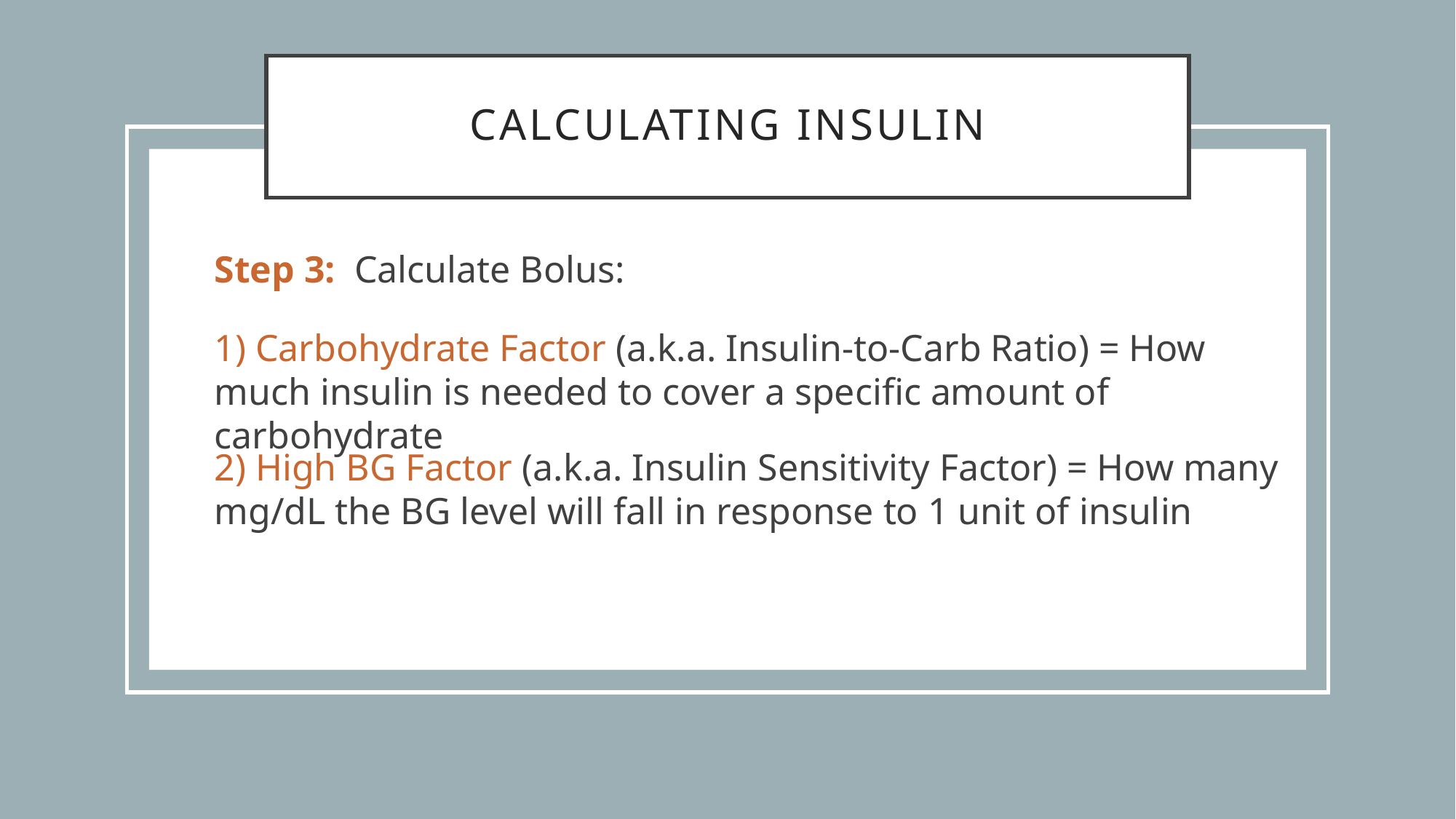

# Calculating insulin
Step 3: Calculate Bolus:
1) Carbohydrate Factor (a.k.a. Insulin-to-Carb Ratio) = How much insulin is needed to cover a specific amount of carbohydrate
2) High BG Factor (a.k.a. Insulin Sensitivity Factor) = How many mg/dL the BG level will fall in response to 1 unit of insulin

## Slide 18
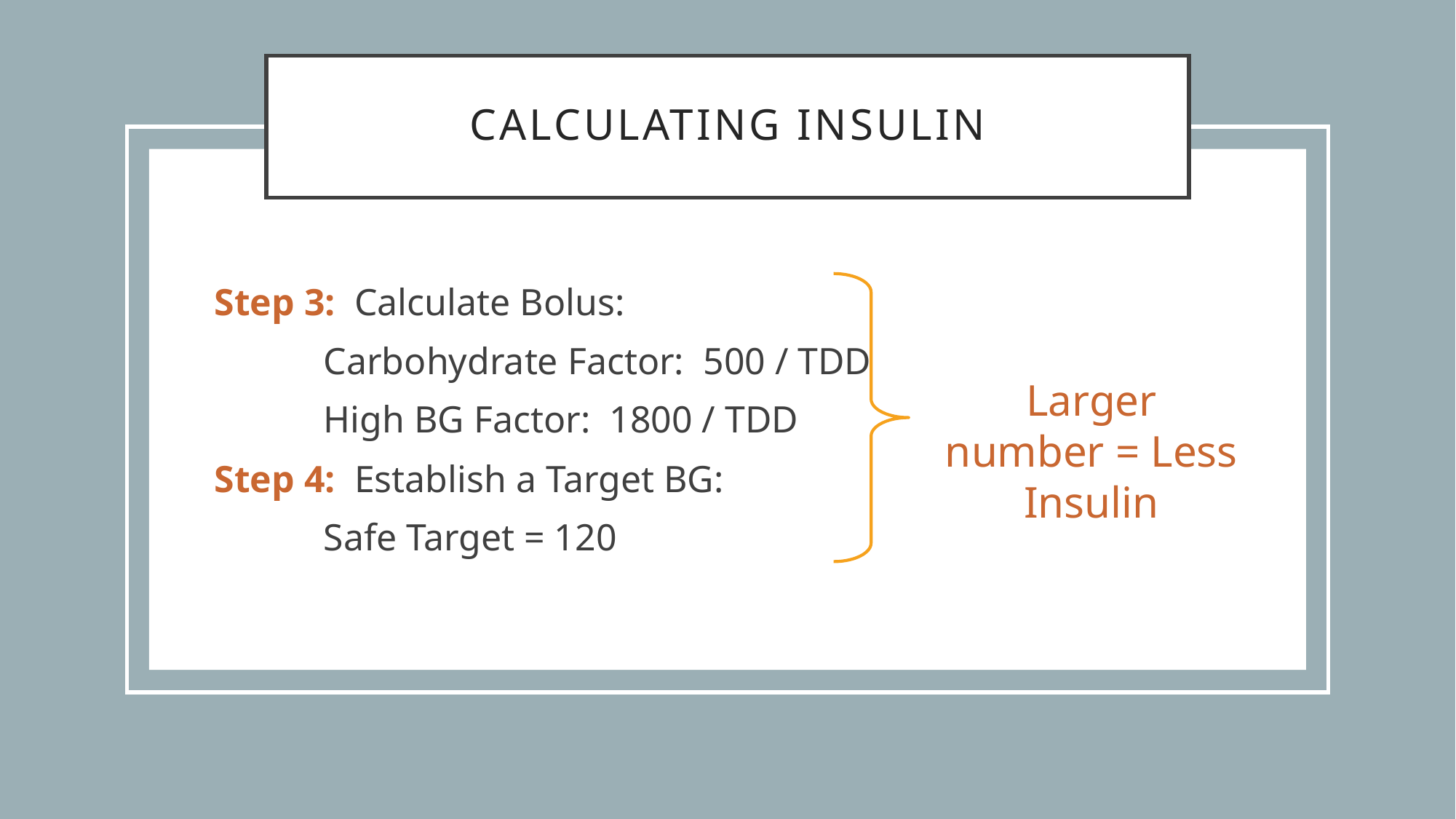

# Calculating insulin
Step 3: Calculate Bolus:
	Carbohydrate Factor: 500 / TDD
	High BG Factor: 1800 / TDD
Step 4: Establish a Target BG:
	Safe Target = 120
Larger number = Less Insulin

## Slide 19
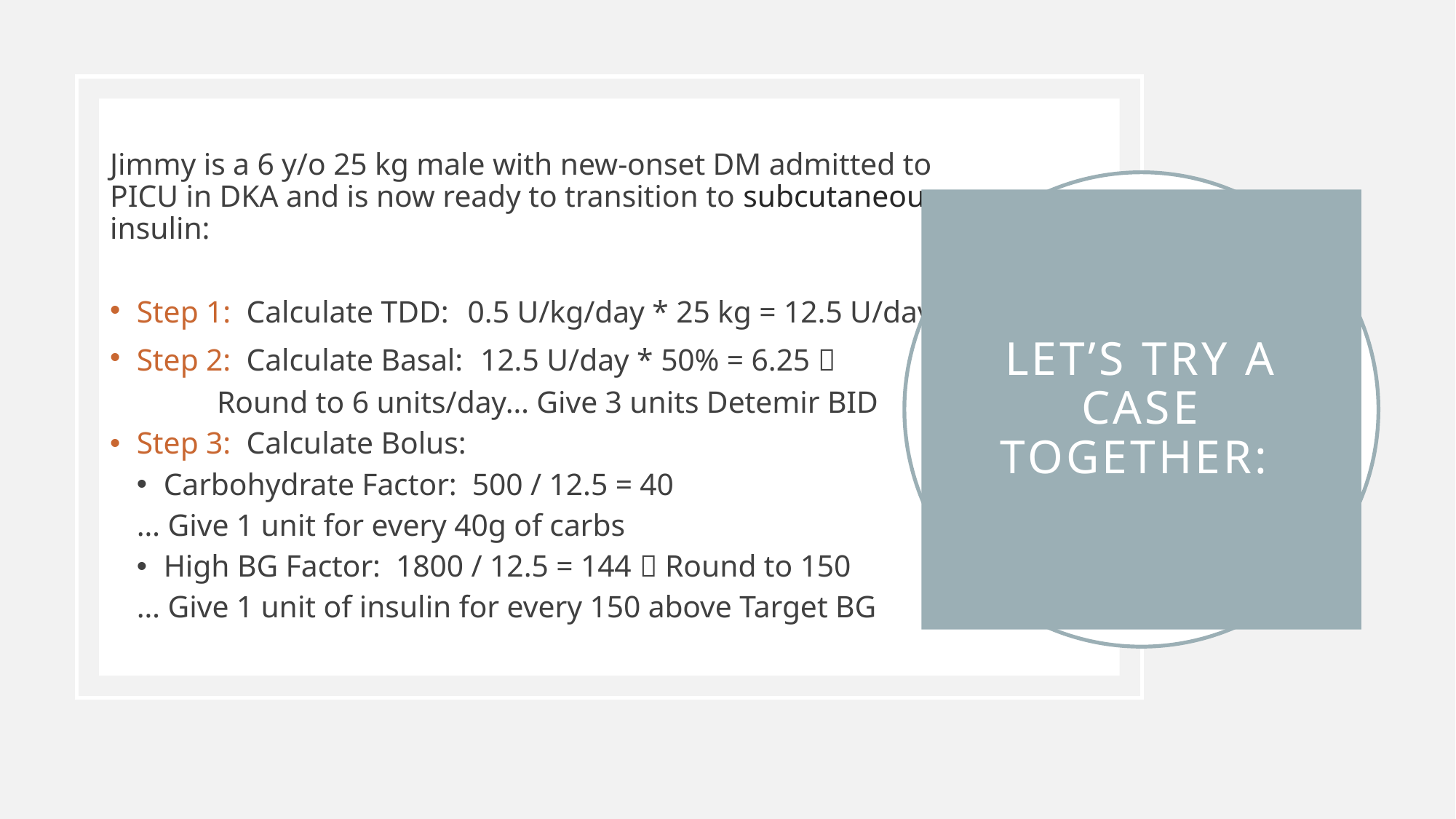

Jimmy is a 6 y/o 25 kg male with new-onset DM admitted to PICU in DKA and is now ready to transition to subcutaneous insulin:
Step 1: Calculate TDD: 0.5 U/kg/day * 25 kg = 12.5 U/day
Step 2: Calculate Basal: 12.5 U/day * 50% = 6.25 
	Round to 6 units/day… Give 3 units Detemir BID
Step 3: Calculate Bolus:
Carbohydrate Factor: 500 / 12.5 = 40
	… Give 1 unit for every 40g of carbs
High BG Factor: 1800 / 12.5 = 144  Round to 150
	… Give 1 unit of insulin for every 150 above Target BG
# Let’s Try a Case Together:

## Slide 20
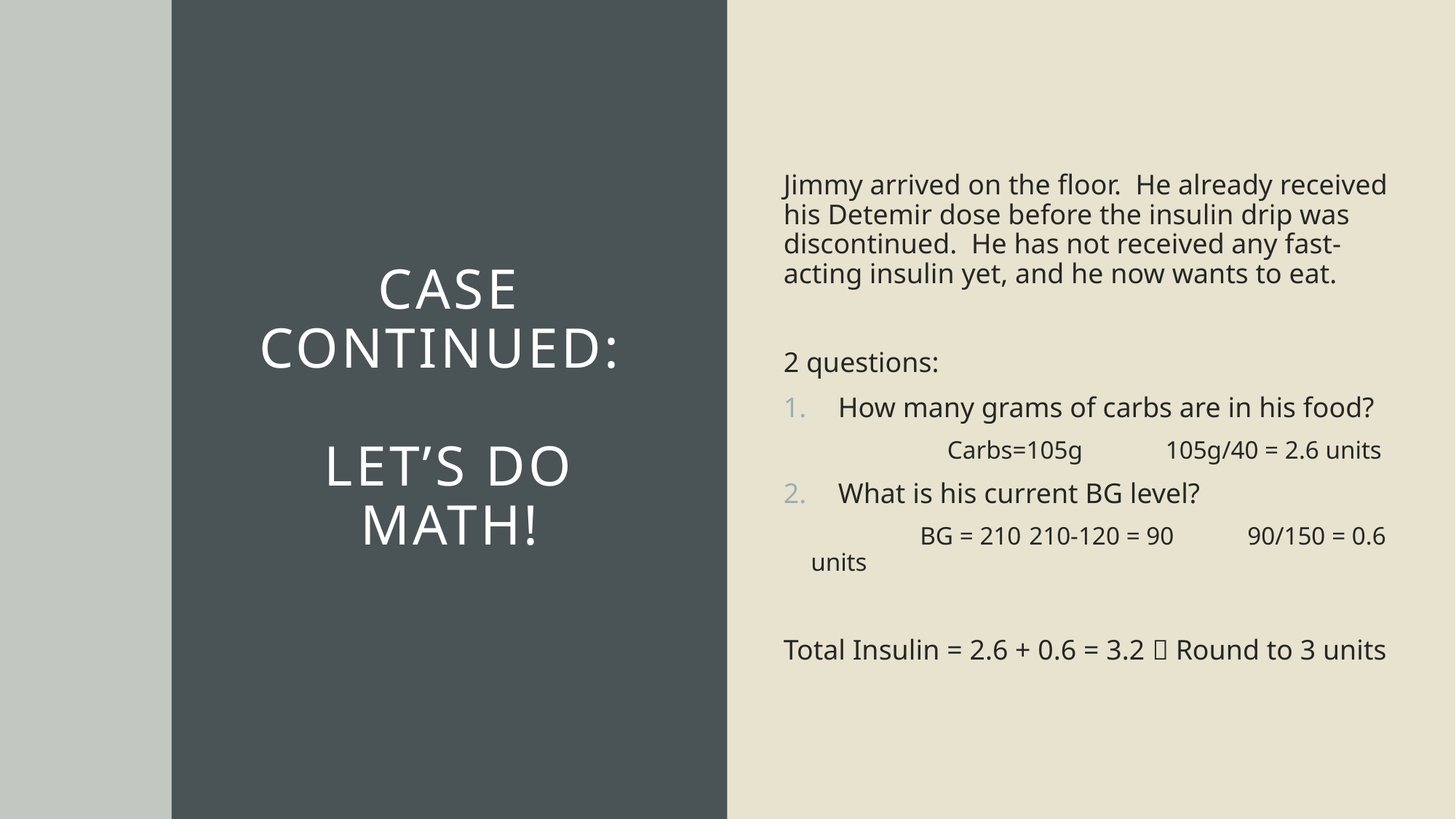

# Case Continued: Let’s Do Math!
Jimmy arrived on the floor. He already received his Detemir dose before the insulin drip was discontinued. He has not received any fast-acting insulin yet, and he now wants to eat.
2 questions:
How many grams of carbs are in his food?
 	Carbs=105g 	105g/40 = 2.6 units
What is his current BG level?
	BG = 210	210-120 = 90	90/150 = 0.6 units
Total Insulin = 2.6 + 0.6 = 3.2  Round to 3 units

## Slide 21
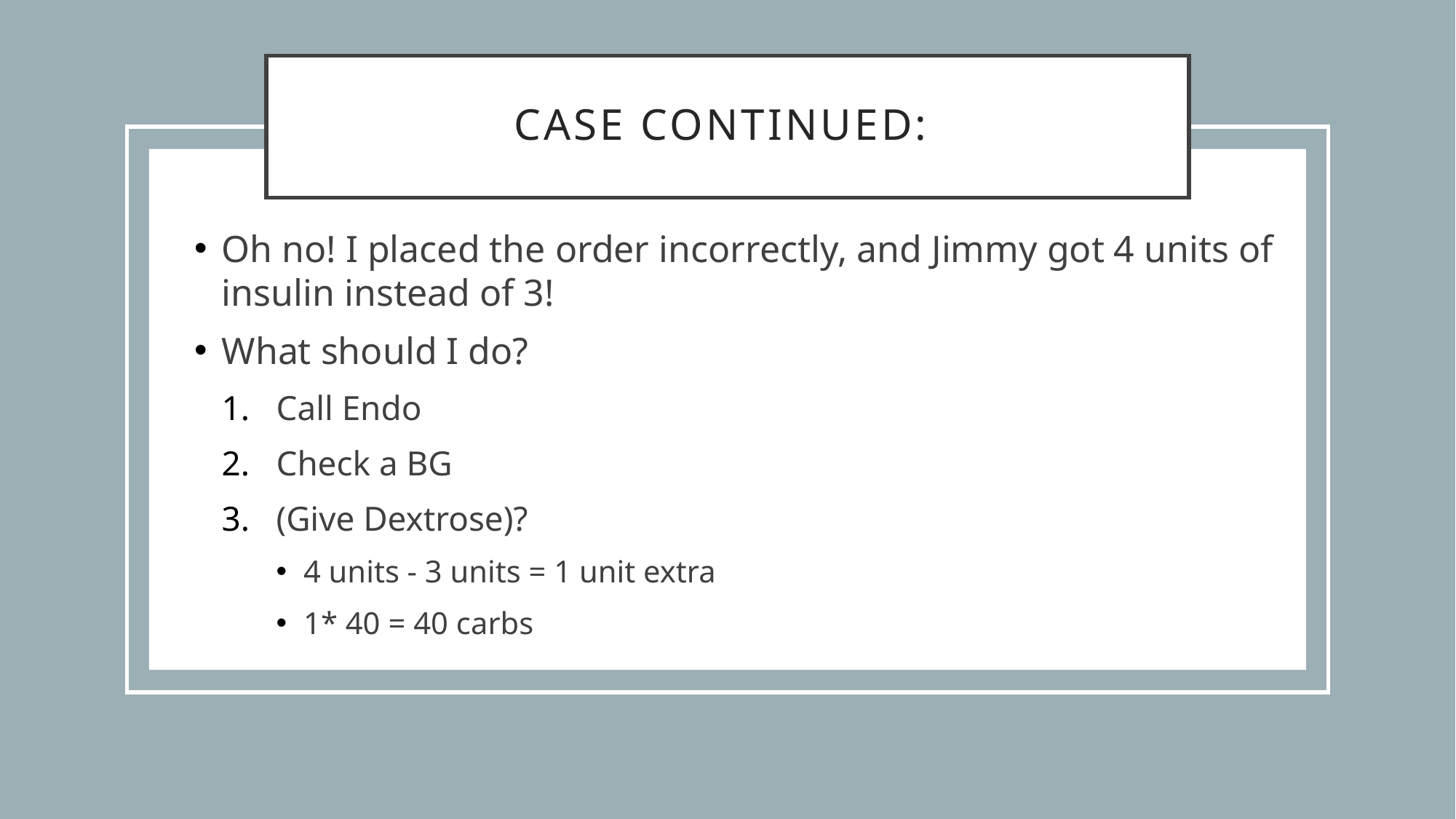

# Case Continued:
Oh no! I placed the order incorrectly, and Jimmy got 4 units of insulin instead of 3!
What should I do?
Call Endo
Check a BG
(Give Dextrose)?
4 units - 3 units = 1 unit extra
1* 40 = 40 carbs

## Slide 22
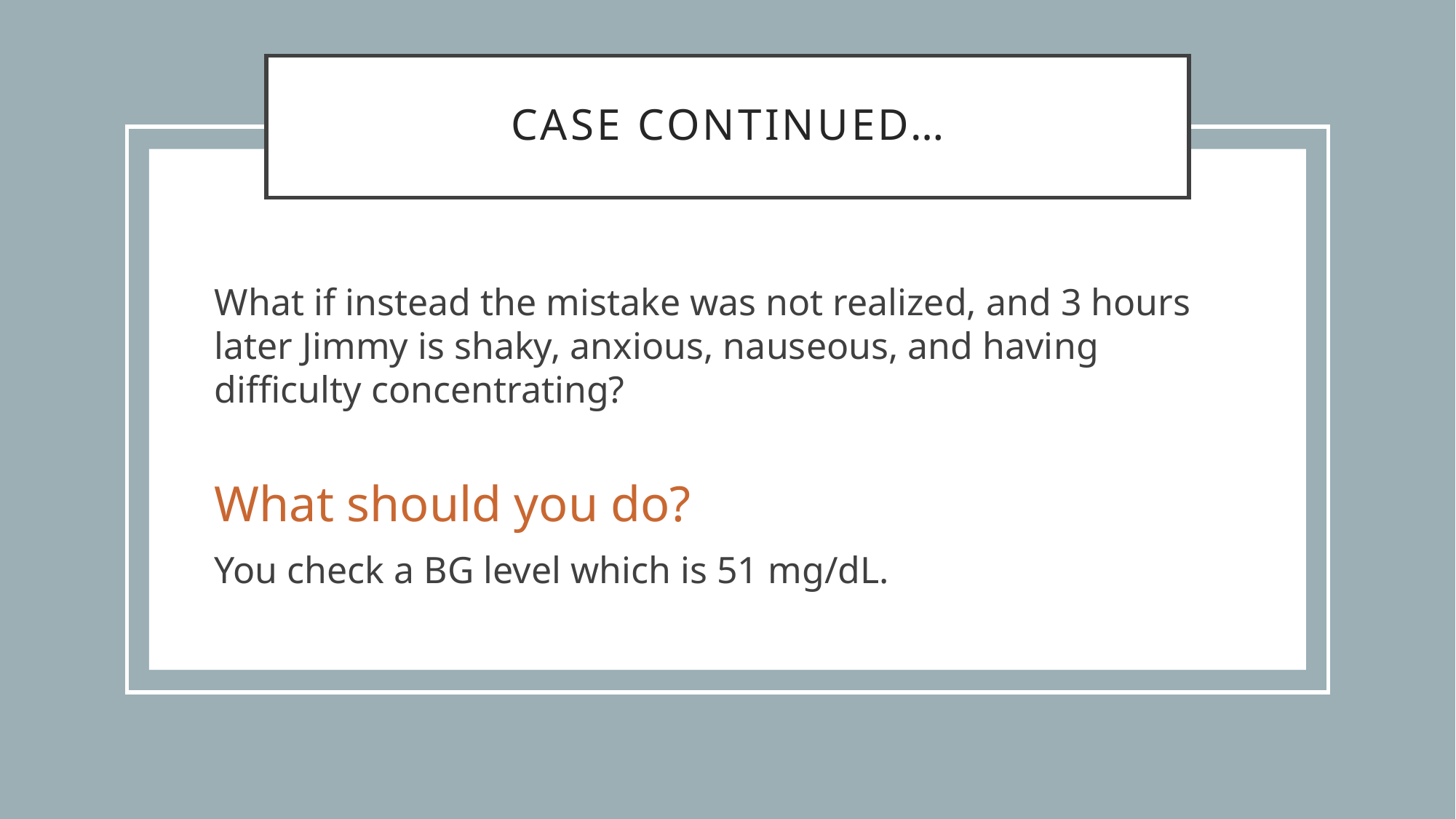

# Case continued…
What if instead the mistake was not realized, and 3 hours later Jimmy is shaky, anxious, nauseous, and having difficulty concentrating?
What should you do?
You check a BG level which is 51 mg/dL.

## Slide 23
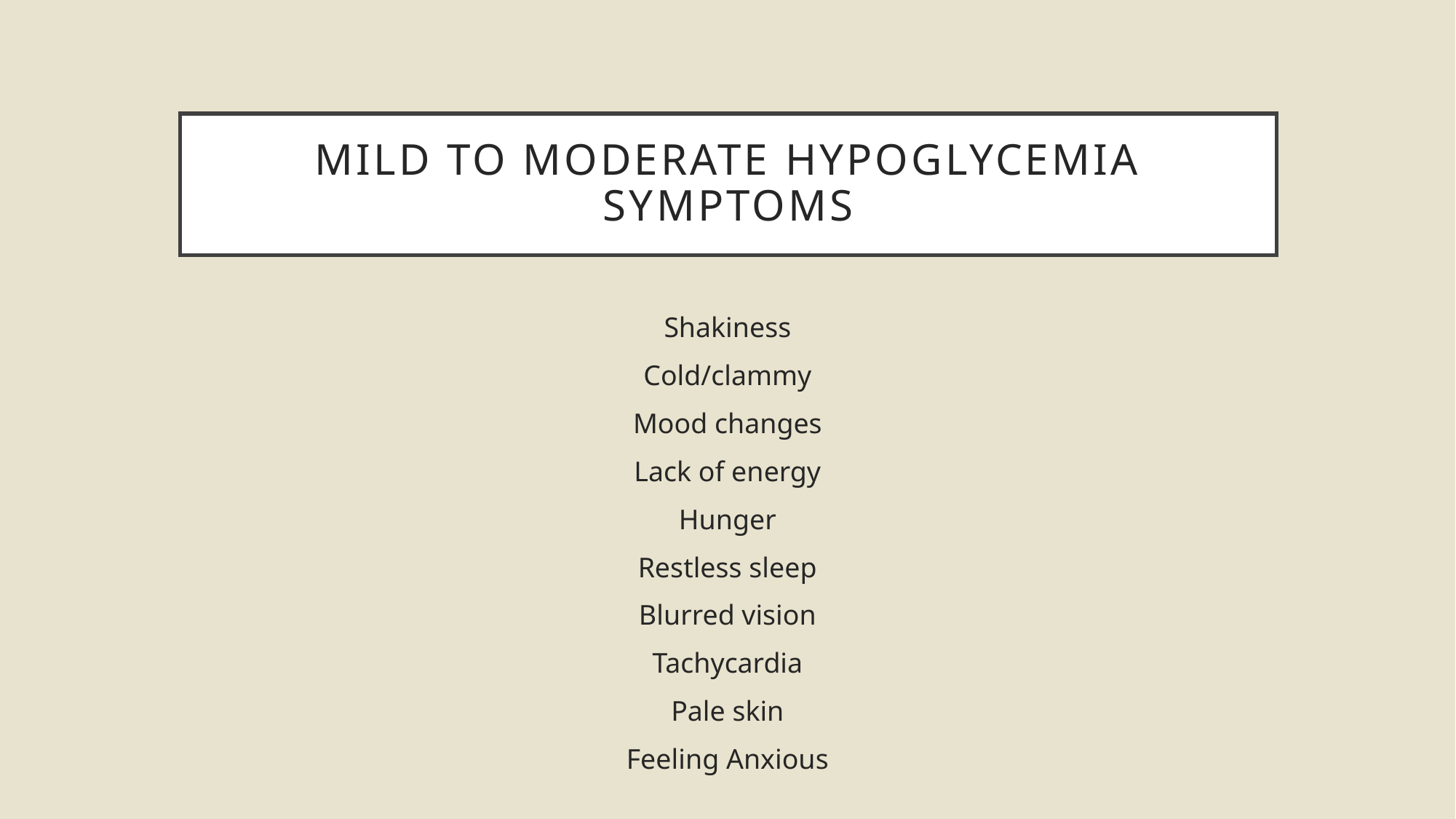

# Mild to Moderate Hypoglycemia symptoms
Shakiness
Cold/clammy
Mood changes
Lack of energy
Hunger
Restless sleep
Blurred vision
Tachycardia
Pale skin
Feeling Anxious

## Slide 24
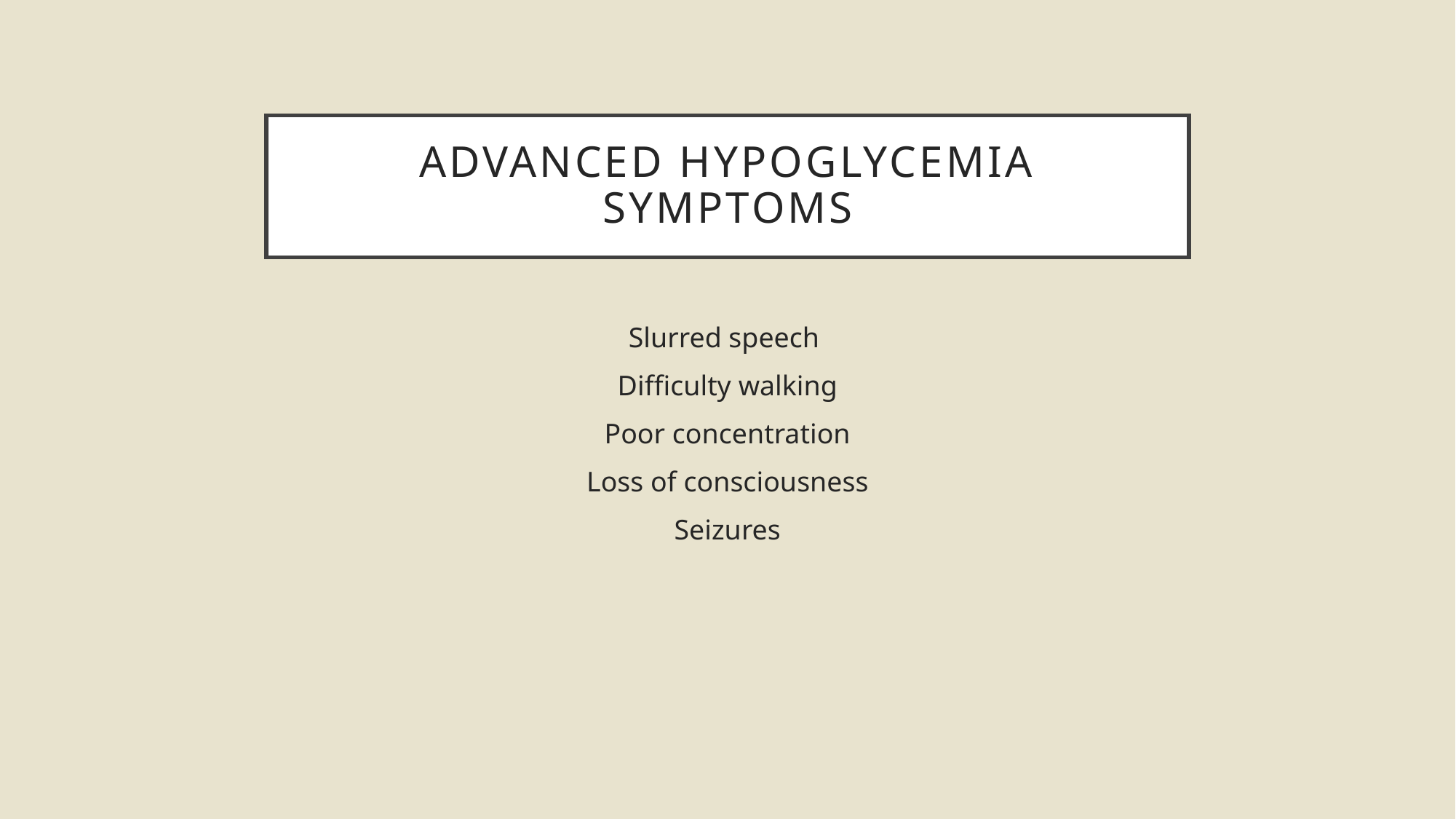

# Advanced Hypoglycemia Symptoms
Slurred speech
Difficulty walking
Poor concentration
Loss of consciousness
Seizures

## Slide 25
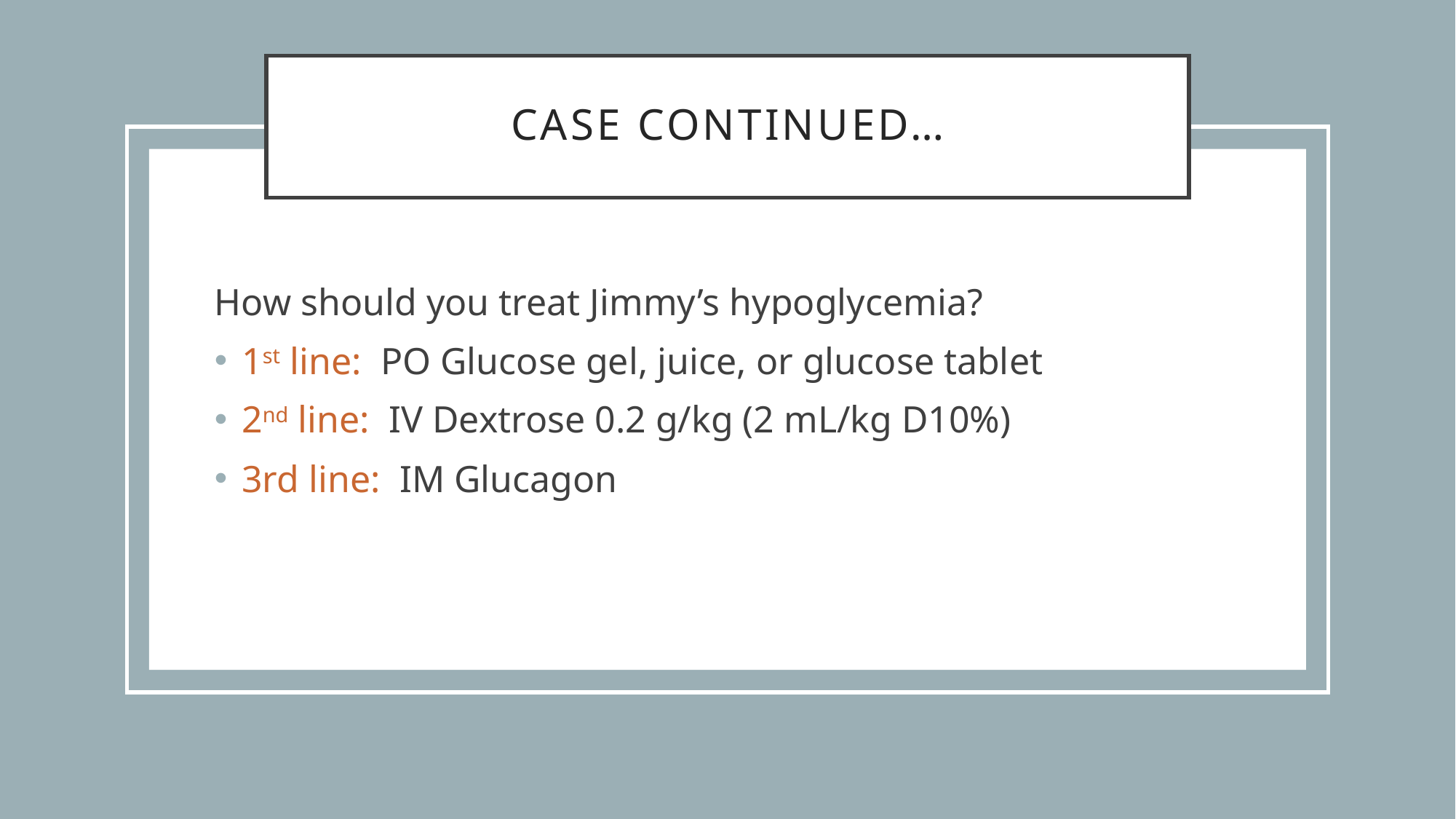

# Case Continued…
How should you treat Jimmy’s hypoglycemia?
1st line: PO Glucose gel, juice, or glucose tablet
2nd line: IV Dextrose 0.2 g/kg (2 mL/kg D10%)
3rd line: IM Glucagon

## Slide 26
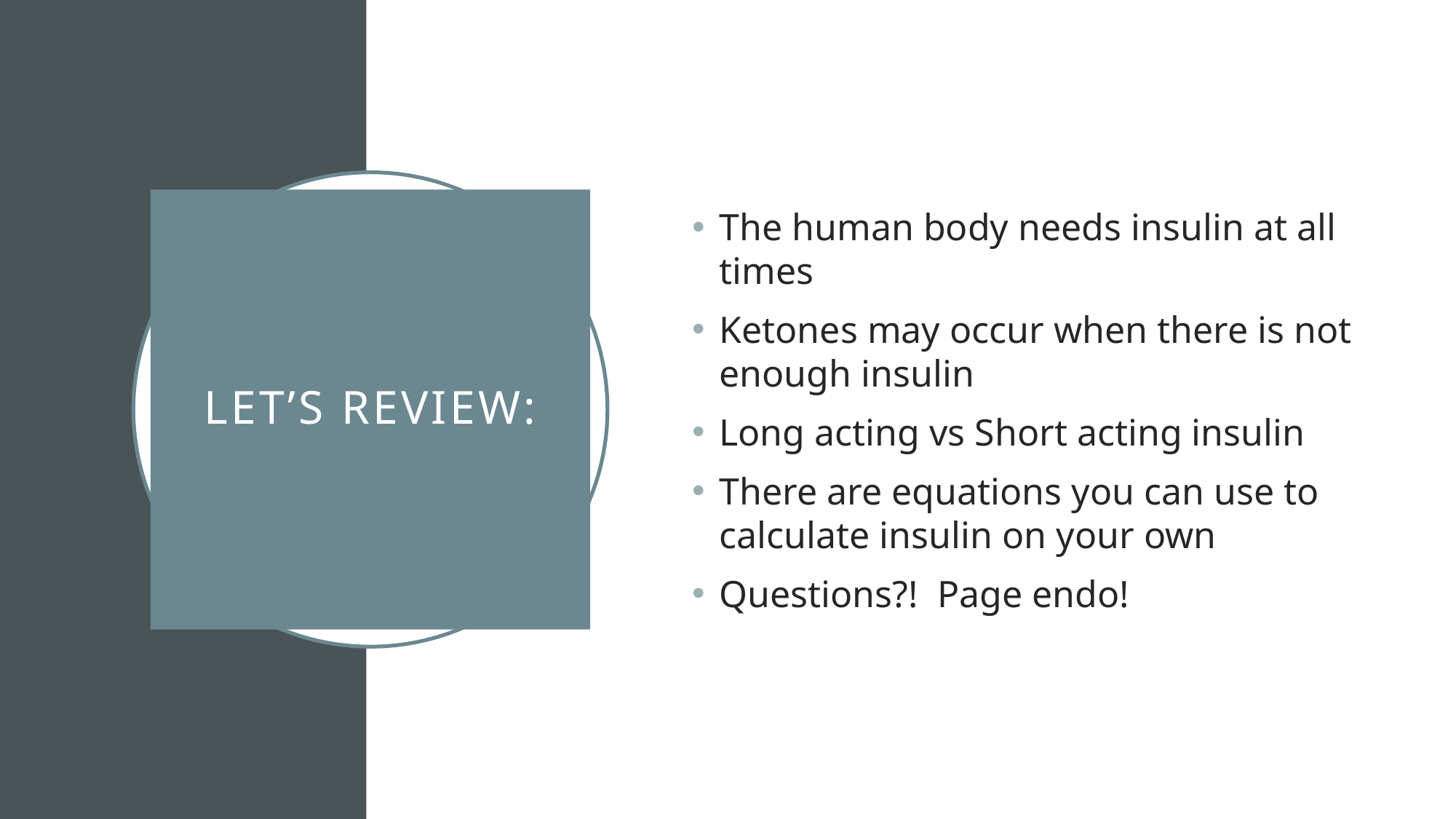

The human body needs insulin at all times
Ketones may occur when there is not enough insulin
Long acting vs Short acting insulin
There are equations you can use to calculate insulin on your own
Questions?! Page endo!
# Let’s Review:

## Slide 27
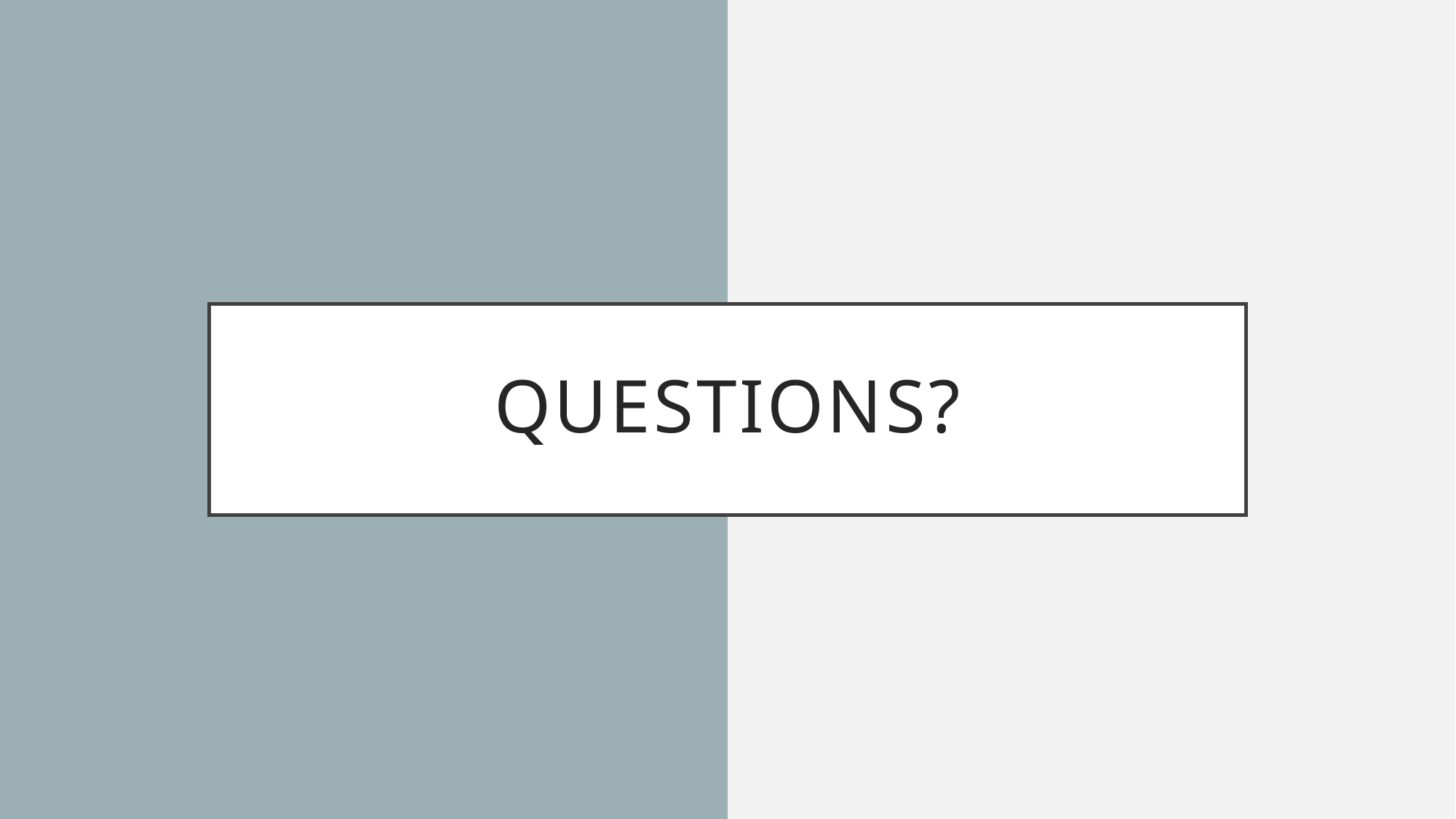

# Questions?
